# Environmental phylogenetics supports a steady diversification of crown eukaryotes starting from the mid-Proterozoic

**DOI:** 10.1073/pnas.2600283123

**Published:** 2026-07-16

**Authors:** Miguel M. Sandin, Phoebe A. Cohen, Hélène Morlon, Fabien Burki

**Affiliations:** ^a^ Department of Organismal Biology, Program in Systematic Biology, Uppsala University, Uppsala 75 236, Sweden; ^b^ Institut de Biologia Evolutiva (Consejo Superior de Investigaciones Científicas-Universitat Pompeu Fabra), Barcelona 08003, Spain; ^c^ Department of Geosciences, Williams College, Williamstown, MA 01267; ^d^ Institut de Biologie de l’Ecole Normale Supérieure, CNRS, INSERM, Université Paris Sciences et Lettres, Paris 75005, France

**Keywords:** macroevolution, diversification, paleobiodiversity, molecular clock, metabarcoding

## Abstract

Molecular clock and preservation of early microfossil assemblages suggest that eukaryotes were present and already diverse more than ~1600 million years ago (Ma). Yet, the earliest identifiable eukaryotic crown group fossil only appeared around 1050 Ma, leaving a ~600 My gap in the Mesoproterozoic during which the evolution and diversification of early eukaryotes remain poorly understood. Here, we infer a timeline of eukaryote evolution using molecular clock and birth–death diversification models, including the large diversity of environmental sequences to take into account the uncultured majority of microbial life. Our analyses, based on a unique dataset of 75,975 nonredundant Operational Taxonomic Units and 77 well-supported fossil calibrations, indicate a steady diversification of crown group eukaryotes during the Proterozoic after the Last Eukaryotic Common Ancestor. We show that Archaeplastida was one of the earliest diversifying supergroups and the most diverse throughout the Proterozoic, suggesting that the first successful plastid endosymbiosis gave Archaeplastida a measurable evolutionary advantage. Discoba, Amoebozoa, and Rhizaria followed Archaeplastida in phylogenetic diversity throughout the Proterozoic, indicating that crown eukaryotes were already thriving in this Era. These results contrast with the common view that the geologically stable Proterozoic Era experienced little eukaryotic evolutionary innovation and indicate that all current supergroups were already established in the Mesoproterozoic.

## Significance

Conventional views portray the Mesoproterozoic (1.6 to 1.0 billion years ago) as a period of biological and geological stasis, in part because the fossil record is sparse and taxonomically ambiguous. Integrating environmental sequencing to obtain species-level molecular phylogenies, molecular clock estimates, and diversification models, we show that crown-group eukaryotes were already taxonomically and ecologically diverse throughout the Mesoproterozoic, revealing complex communities well before the Neoproterozoic radiation. The results lead us to hypothesize that endosymbiosis and other biotic interactions, including predation, were major early drivers of eukaryotic diversification. These findings revise interpretations of Proterozoic ecosystems and provide a molecular framework that helps reconcile fossil evidence with the timing and mechanisms of early eukaryotic evolution.

The origin of eukaryotes is a major evolutionary transition in the history of life that has shaped our planet in countless ways. Today, eukaryotes are found in virtually every environment on earth, ranging from tiny single-cells to the largest organisms to have ever lived. Although most attention is paid to macroeukaryotes, it is the microbes that have dominated eukaryote evolution for most of their history (1, 2). These microbial eukaryotes continue to make up the vast majority of diversity across the eukaryotic tree of life, having evolved into an incredible diversity of forms, lifestyles, and functions (3). In recent years, we have learned a great deal about the origin of eukaryotes from the merging of Archaea and Bacteria (e.g., refs. 4–8), but the early history of eukaryotes after their last common ancestor (LECA) remains poorly understood. In particular, we know little about the diversification dynamics of the main eukaryotic supergroups following their origin.

The oldest unambiguous eukaryotic fossils appeared ~1750 Ma with acritarchs inferred to possess complex features such as a cytoskeleton and endomembrane system (9). By ~1640 Ma, eukaryotes were taxonomically more diverse corresponding to simple multicellular and possibly photosynthetic forms in shallow marine environments (10–15). While the acritarch fossil record has become richer and more informative about the deep history of eukaryotes in recent decades, it can only serve as a minimum time bound for the origin of total eukaryotic clade (i.e., stem and crown groups). Beyond the morphological fossil record, size-based ecosystem modeling suggests a complex eukaryotic community from ~1700 Ma onward, composed of heterotrophs, autotrophs, and even mixotrophs (16). In addition, the rock record points to abundant sterol precursors of possible eukaryotic origin (protosteroids and ursteroids) during the late Paleoproterozoic and Mesoproterozoic (17). However, it was not until the late Mesoproterozoic and early Neoproterozoic that undisputed fossil representatives of extant eukaryotic groups (crown groups) appeared. These fossils include *Bangiomorpha*, commonly accepted as a 1050 Ma red algal (18), and the 950 Ma green algal *Proterocladus* (19), both members of the supergroup Archaeplastida and evidence of its increasing ecological and taxonomic diversity. Therefore, there is a ~600 My gap in the fossil record between the oldest known total group fossil eukaryotes and the ecological rise of the first crown groups, during which time the diversification dynamics of eukaryotes remain poorly understood.

Complementary to evidence from the sedimentary rock record, molecular clock analyses and phylogenetic diversification models allow us to infer the divergence times and diversification of species across phylogenetic clades, particularly in groups with poor fossil records. Current estimates based on phylogenomics and advanced molecular clock analyses indicate that crown eukaryotes diversified ~1850 to 1600 Ma (8, 20, 21), or possibly earlier (22), in good agreement with the Paleoproterozoic fossil record reporting complex eukaryotic (stem or crown) communities. While rich in genomic data, these molecular clock analyses suffer from a relatively poor representation of the massive lineage diversity of microeukaryotes that has been revealed by environmental sequencing (23). This environmental diversity has not yet been used to study diversification patterns across all eukaryotes and the few phylogenetic diversification analyses that have included environmental diversity focused mostly on specific taxonomic groups such as diatoms (24) or ciliates (25). As a consequence, we know little about the diversification history of early eukaryotes. While diversity dynamics estimated from molecular phylogenies are probable reconstructions, with associated uncertainties depending on the confidence we have in phylogenetic data, as well as on the adequacy and identifiability of the diversification models used (26–31), they provide valuable estimates that are useful to contrast with those obtained from fossil data (e.g., refs. 32–34). For systems whose fossil record is sparse and cryptic, such as bacteria and the early eukaryotes studied here, such phylogenetic analyses are the only method of studying diversification over geological time (35).

Here, we used a molecular approach to studying the diversification dynamics of all major eukaryotic groups from the Proterozoic until today. We built a unique phylogenetic dataset of 75,975 nonredundant Operational Taxonomic Units (OTUs), integrating 77 well-supported fossil calibrations and information from the large environmental diversity with long-read metabarcoding data of the near-full eukaryotic ribosomal operon (18S-28S rDNA) and curated 18S rDNA sequences. Our analyses provide phylogenetic evidence of the complexity of eukaryotic communities during the Mesoproterozoic and show that crown group eukaryotes were taxonomically diverse nearly 800 My before direct evidence of morphological diversification in the fossil record. These results suggest that crown group eukaryotes were not only present but diversifying and therefore active players in eukaryotic communities ever since their last common ancestor. Altogether, this study brings an important molecular perspective in eukaryotic paleoecology and complements interpretations of the fossil record.

## Results

### Timetree of Eukaryotes Including the Vast Environmental Diversity.

In order to include as much diversity as possible in our analyses, we took advantage of the very large collection of ribosomal DNA (rDNA) available for microbial eukaryotes (protists), focusing on environmental sequences. Our dataset combines long-read metabarcoding data of the 18S-28S rDNA, which provide improved phylogenetic signal over classical short-read data, with available 18S rDNA reference sequences to obtain 75,975 curated nonredundant OTUs spanning the broad eukaryotic diversity. Topological constraints taken from a consensus of published phylogenomic studies were applied to fix ambiguous deep nodes using rDNA alone (*SI Appendix*, Fig. S1). A range of 32 phylogenetic trees were reconstructed using alternative alignments and evolutionary models to take into account phylogenetic uncertainties (*SI Appendix*, Fig. S2).

**Fig. 1.**
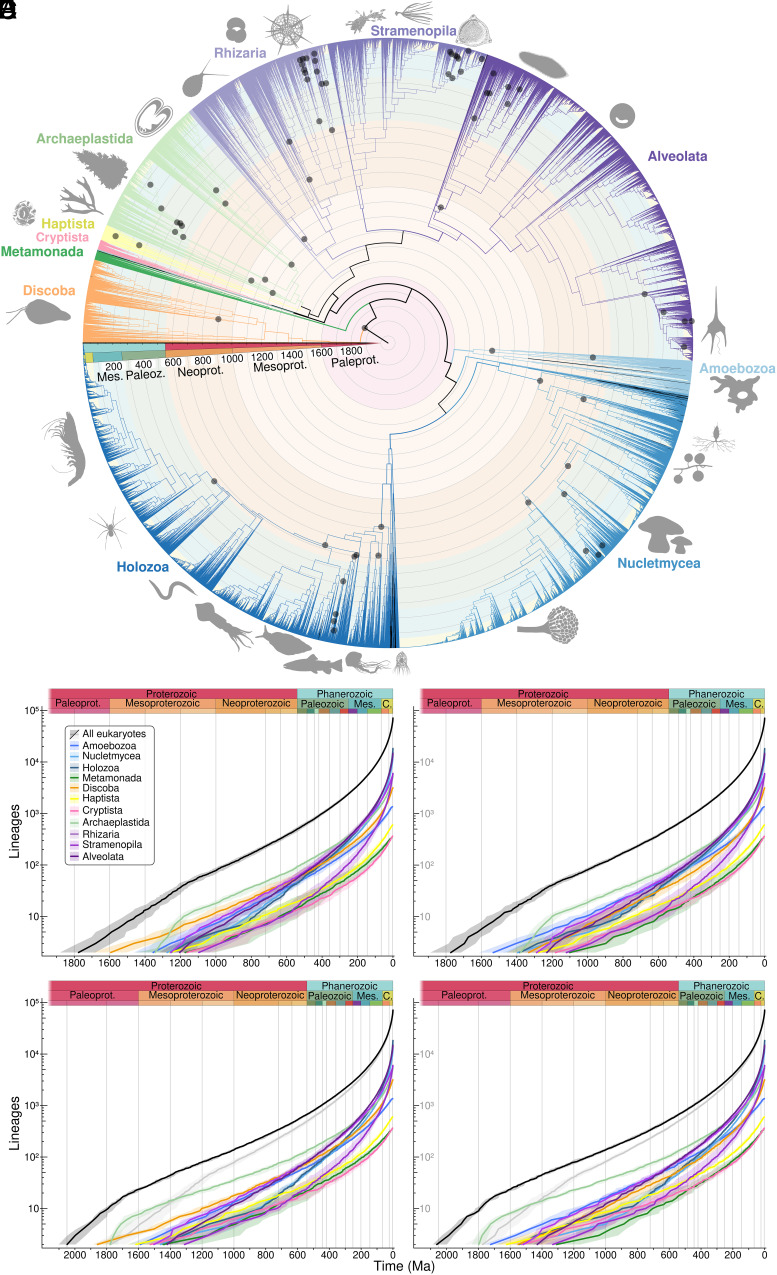
(*A*) A time-calibrated phylogenetic tree of extant eukaryotes, rooted in Discoba, containing 71,138 nonredundant 99% OTUs of the rDNA (5794 aligned positions) selected from the total 32 time-trees based on the highest likelihood. Colored branches represent different eukaryotic supergroups (only those with more than 300 tips are named) and background color different geological eras. Black dots on nodes highlight calibrated nodes. Silhouettes represent major branches of morphologically described diversity for visual guidance (e.g., The fish on the lower left, *Salmo trutta*, represents all Chordate lineage, from other fishes and sharks to humans, birds, and amphibians). (*B* and *C*) Summary of the LTT plots across supergroups based on calibrated trees rooted at Discoba (*B*) and Amorphea (*C*). Lines represent the median and shaded areas the 90% centered percentile, among the 32 trees with median ages (see *SI Appendix*, Fig. S4 for a summary of all independent time-trees with their corresponding Highest Posterior Densities ages). (*D* and *E*) Same as (*B* and *C*), but including the dubious *Rafatazmia* calibration for Archaeplastida at 1600 Ma, and using 10 randomly selected trees.

Given existing doubts on the position of the root of the eukaryotic tree, we tested rooting on Discoba or Amorphea, corresponding to two main alternative roots for the eukaryotic tree (36–38). The trees were time-calibrated using a main set of 77 diverse accepted fossil calibrations, referred to as MC01 (*SI Appendix*, Fig. S3 and Dataset S1). The 95% Highest Posterior Densities -HPD- of the estimated dates of LECA and the first divergence of the supergroups reveal relatively large uncertainty (*SI Appendix*, Fig. S4). The median estimated dates, summarized as Lineages Through Time (LTT) plots, were otherwise largely consistent across the 32 timetrees (see Fig. 1*A* for the best log likelihood scoring tree). The root position had a minimal effect on the overall ages throughout the trees (*SI Appendix*, Fig. S4), with a median root age of 1776 Ma (and maximum and minimum medians of 1897 and 1670 Ma, respectively) for the Discoba root (Fig. 1*B*) and 1773 Ma (1934 to 1703 Ma) for the Amorphea root (Fig. 1*C*). Below we arbitrarily focus on the Discoba-rooted trees for all subsequent results. The first observed supergroup divergence occurred in Discoba at 1594 (1726 to 1471) Ma, followed by Amoebozoa (1354; 1493 to 1257 Ma) and shortly after Archaeplastida (1343; 1421 to 1266 Ma) (Fig. 1*B*, but see also Fig. 1*C* and *SI Appendix*, Fig. S4 for different rooting scenarios and HPDs). All the other supergroups originated within a ~200 My span during the Mesoproterozoic starting ~1275 Ma.

The impact of disputed fossil calibrations on our inferred dates was assessed by using three additional calibration sets (MC02-04, see *Material and Methods* for a rationale on the choice of the fossil calibrations). The effect of these uncertain fossils was variable depending on their age and taxonomic assignments. The Proterozoic *Rafatazmia* fossil (MC02), initially attributed to crown Rhodophyta (39) but later questioned (18, 40), had a large effect by pushing back the timing of LECA (the first divergence of eukaryotes) to 2054 (2104 to 1967) Ma (Fig. 1 *D* and *E*, MC02). Conversely, when considering *Bangiomorpha* as crown Rhodophyta (21, 40) instead of Bangiophyceae, as in the main calibration set (i.e., MC03 calibrates the group Rhodophyta whereas MC01 the subclade Bangiophycea), had no detectable effects (*SI Appendix*, Fig. S5, MC03). Finally, not considering the calibration corresponding to the first divergence of crown diatoms, as recently argued (41), had also no significant impact (*SI Appendix*, Fig. S5, MC04).

### Estimating Total Extant Diversity of Eukaryotes.

Phylogenetic diversification analyses require an estimate of the sampled fraction of the hypothetical total extant diversity of the taxonomic groups under scrutiny to reliably estimate speciation and extinction rates (42, 43). Although we sampled the broadest available extant eukaryotic diversity, from high-rank taxa to species level, encompassing both morphologically described species and the majority of uncultured environmental sequences, we may still be missing significant portions of the total diversity. We implemented two different approaches relying on species abundance distributions to estimate the fraction of the total eukaryotic diversity included in our data (*Material and Methods*). On average, our phylogenetic trees contained between 50% and 77% of the hypothetical total eukaryotic diversity as defined by OTU clustering at 99% of the 18S rDNA, with the lower sampling fraction fitting slightly better the observed data (*SI Appendix*, Fig. S6). In addition, the sampling fraction is not uniformly distributed across the tree. While groups such as Nucletmycea and Holozoa were relatively well sampled, others such as Rhizaria, Alveolata, and Cryptista were less well represented. In general, these groups also have a much lower proportion of morphologically described species than environmental OTUs, as compared to groups such as Holozoa (*SI Appendix*, Fig. S7).

### Diversification of Eukaryotes.

**Fig. 2.**
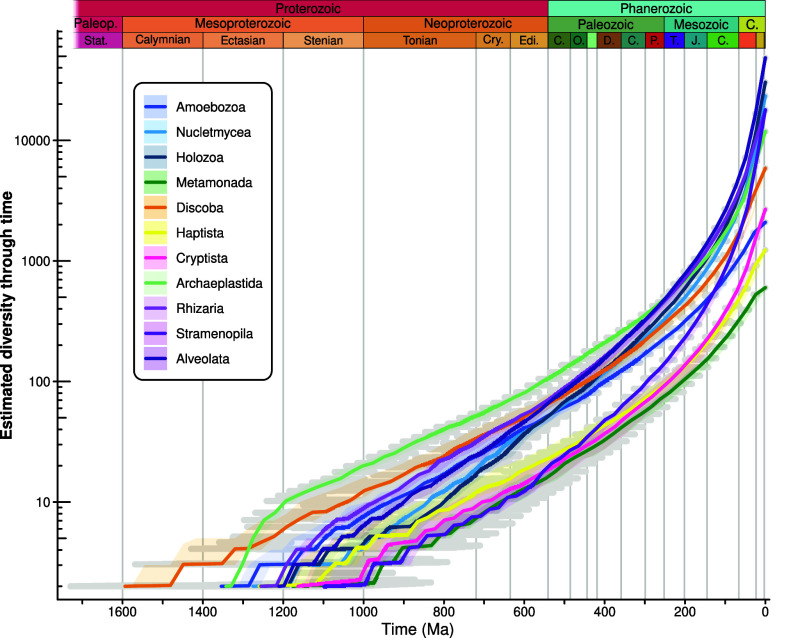
Summary of the estimated DTT plots obtained with ClaDS for the Discoba rooted scenario assuming the lowest range of sampling fractions (see *SI Appendix*, Fig. S8 for the Amorphea rooted scenario and the other sampling fractions). Lines represent the median and shaded areas the 90% Highest Posterior Density (HPD), respectively, of all independent DTT. Gray horizontal lines represent the 90% HPD of the time scale.

While insights into the phylogeny and early evolution of eukaryotes have greatly improved in the last 20 y, the patterns of diversification across the eukaryotic tree, especially during the Proterozoic, have been much less extensively explored. Using our taxon-rich dataset, we analyzed the diversification of each supergroup using the ClaDS phylogenetic birth–death model (44, 45), with data augmentation to estimate Diversity Through Time (DTT, Fig. 2 and see *SI Appendix*, Fig. S8 for other rooting and sampling fraction scenarios), as well as mean speciation (λ) and extinction rates (μ) through time, from which net diversification rates can be estimated (r = λ − μ, *SI Appendix*, Fig. S10). This model accounts for rate heterogeneity across lineages, making it suitable for large scale phylogenetic diversification analyses.

Archaeplastida and Rhizaria accumulated diversity rapidly shortly after their origin, before slowing down in the late Mesoproterozoic and later increasing again in the Phanerozoic and in the mid-Neoproterozoic, respectively (Fig. 2). Archaeplastida experienced the fastest early accumulation of lineages following their origin at 1343 (1391 to 1276) Ma and for the longest time period, resulting in the highest diversity between ~1300 Ma until the early Mesozoic (Fig. 2). This rapid accumulation of lineages is also seen within the two most speciose archaeplastid groups (Chloroplastida and Rhodophyta) (*SI Appendix*, Fig. S9), suggesting a general trend in Archaeplastida. In addition, this trend is also recovered when using the calibration set including *Rafatazmia* (MC02, *SI Appendix*, Fig. S8). The supergroups other than Archaeplastida, and to some extent Rhizaria, first accumulated diversity relatively slowly but steadily starting in the Proterozoic, before an acceleration toward present (Fig. 2).

The trends in diversification rates are remarkably consistent across groups showing a steady increase toward the present (*SI Appendix*, Fig. S10). This is reflected in the ClaDS trend parameter α, and heterogeneity parameter σ, which together predict that daughter lineages have on average a higher speciation rate than their parents (m > 1, *SI Appendix*, Fig. S11). Although extinction rate estimates are overall low compared to speciation, most differences in diversification rates are due to differences in extinction rates rather than speciation rates, with supergroups with a lower OTU richness (i.e., Cryptista, Haptista, and Metamonada) showing extinction rates several orders of magnitude higher than supergroups with high OTU richness (*SI Appendix*, Fig. S10). While undersampling may underestimate extinction rates [by diminishing the “pull of the present” (26) from which information on extinction is gathered, see *Material and Methods*], all supergroups showed a comparable fit of the sampling fraction (*SI Appendix*, Fig. S6). Given that this sampling fraction is not related to the OTU richness of the supergroup, a comparable sampling bias is expected across all supergroups.

### Speciation Rate Shifts across the Tree.

**Fig. 3.**
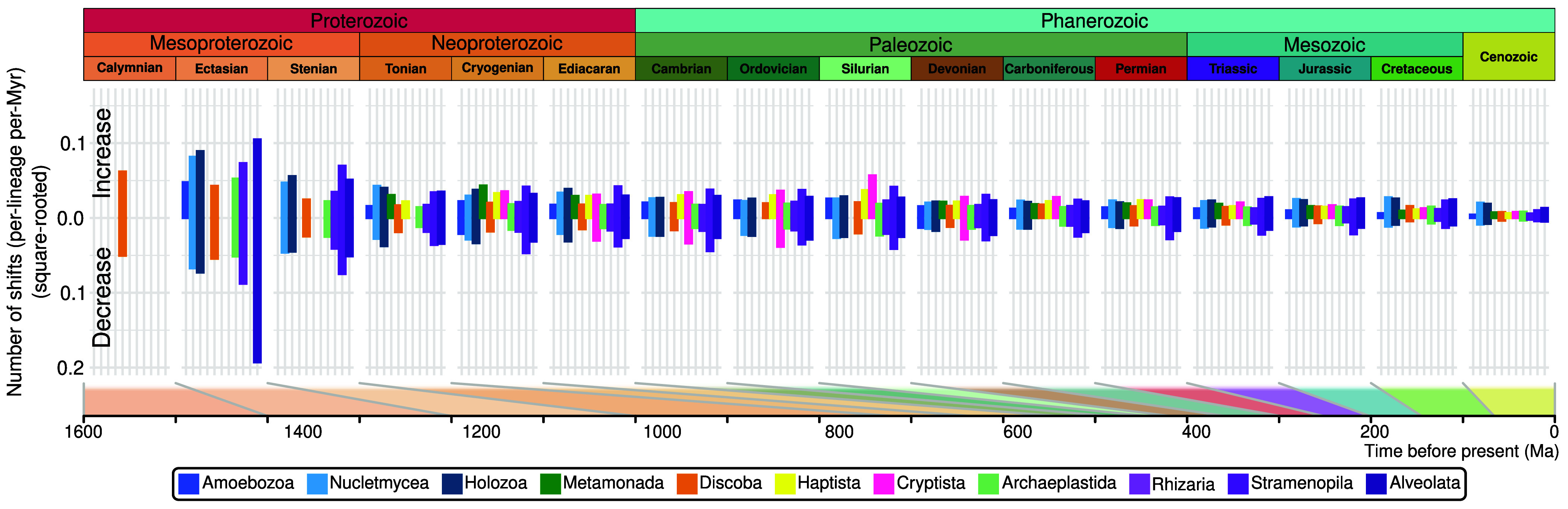
Proportion of shifts in speciation rate (squared rooted) across geological time, under the Discoba rooted scenario and assuming the lowest range of sampling fractions (see *SI Appendix*, Fig. S13 for the Amorphea rooted scenario and the other sampling fractions). “Increase” indicates a shift toward a higher speciation rate and “decrease” indicates a shift toward a lower speciation rate. The proportion of shifts was calculated by dividing the total number of shifts per time period (*SI Appendix*, Fig. S12) by the total branch-length within the given time period.

A shift increase of the speciation rate on specific branches of the tree can result from key innovations, specific adaptations, or variations in biotic and/or abiotic conditions that promote speciation. Similarly, a shift decrease can arise for example if changes in environmental conditions impede speciation. We estimated speciation rate shifts using BAMM (*Material and Methods*) for all different supergroups within all 32 timetrees independently for our main calibration set (MC01), and 10 random trees for MC02. At face value, most identified shifts in the speciation rate occurred closer to the present (*SI Appendix*, Fig. S12), as expected based on the fact that there are more lineages, cumulating more evolutionary time, close to present in phylogenetic trees. However, when correcting the number of shifts by the cumulative time during which they had the opportunity to occur, measured as the total branch-length within geological time periods, the frequency of speciation rate shifts is instead slightly reduced toward present in all supergroups (Fig. 3 and see *SI Appendix*, Fig. S13 for other rooting, sampling fraction scenarios and calibration sets). The most marked decrease in the frequency of shifts occurs from the Mesoproterozoic to the Neoproterozoic, going from a median 4.9 10^−4^ shifts per-lineage per-Myr to 1.74 10^−4^. After that, the frequency of shifts stays relatively constant from the Neoproterozoic through the Paleozoic. In most supergroups we inferred a higher proportion of speciation rate increases than decreases (Fig. 3 and *SI Appendix*, Fig. S13), further supporting the tendency for an increase in diversification rates through time found with ClaDS, and suggesting that diversity “begets” rather than “impedes” more diversity. In contrast, Rhizaria stands out as experiencing the highest proportion of diversification rate decays during the Proterozoic and Phanerozoic, followed by Stramenopila and Alveolata (Fig. 3 and *SI Appendix*, Fig. S13). Overall, the mild decline in rate shifts over time suggests that lineages experienced relatively more key innovations or adaptations that modified their speciation rates early in their evolutionary history.

## Discussion

In this work, we have integrated phylogenetic information from both environmental and reference rDNA sequences into the most taxon-rich molecular clock and diversification analysis across eukaryotes to date. Our analyses place the Last Eukaryotic Common Ancestor (LECA) at ~1775 Ma, in agreement with the first unequivocal eukaryotic fossils (9–11, 46) and other molecular dating analyses of eukaryotic origin [(8, 20, 21, 47, 48); *SI Appendix*, Fig. S14]. Including older but debated calibrations for crown eukaryotes alters the timing of LECA. Specifically, if Rhodophytes are as old as 1600 Ma as implied by the multicellular fossil *Rafatazmia* (39), our analyses shifted the origin of LECA to the mid-Paleoproterozoic (~2054 Ma). Recent phylogenomic analyses indicated a Paleoproterozoic appearance of Archaeplastida (22) even without the *Rafatazmia* calibration (49–51), and thus an earlier origin of LECA and some crown eukaryotes is not without molecular support.

Beyond *Rafatazmia*, whose age and taxonomic assignment to a crown group have been questioned (18, 40), rich and complex assemblages of eukaryotes have been described from the late Paleoproterozoic, including both single-cell fossils from at least ~1750 Ma (9) and simple multicellular organisms at ~1640 Ma (13–15). These fossil data suggest that eukaryotes were present and well established by the late Paleoproterozoic, but a major unanswered question in paleobiology is whether these early fossils may be unidentified members of crown eukaryote groups, thus bridging the gap between LECA (inferred here at 1775 Ma) and the ecological rise of extant supergroups during the late Proterozoic. A temporal offset between the fossil record and molecular dating is to be expected given the sparse Proterozoic fossil and rock record (52), leading to an amplified “Sppil-Rongis” effect (53) which states that incompleteness in the fossil and rock record will necessarily mean that the oldest known fossil in a clade will always be younger than the true clade origination date. This effect is well characterized for much more recent animal and plant fossils (54–56) but is likely even more pronounced for older protist forms that may lack fossilized and/or recognizable structures. While our molecular timing and diversification analyses inferred from only extant taxa cannot place enigmatic acritarchs within a eukaryotic phylogeny, they can help us better understand the evolutionary dynamics of the major eukaryotic groups following their appearance.

Clearly identifiable crown group fossils or crown group biomarkers (i.e., sterols) only appear in the latest Mesoproterozoic. Yet, our results show that most eukaryotic supergroups originated earlier. In addition to the “Sppil-Rongis” effect, the sterol record may suffer from taphonomic biases (57); it is therefore possible that crown group eukaryotes were confined to specific environments where they diversified, but left limited fossil and biomarker evidence behind at a global scale, as suggested by fossil-based diversification modeling (46, 58). Coastal environments, for example, were likely oxygenated enough to sustain the development of eukaryotes since the Great Oxidation Event ca. 2.4 Ga (59) and consistently supported high richness, morphological complexity, and even habitat specialization (60, 61). We speculate that these relatively stable and productive coastal ecosystems may have provided the necessary conditions for both the origin of eukaryotes and their initial gradual diversification into the distinct supergroups reported in our analyses, as supported by fossil assemblages (62). However, speciation rates of the different supergroups remained relatively low during the Proterozoic compared to more recent times. Such slow, but substantial, initial diversification might be related to competition with other contemporary lineages, including stem and crown species, as previously suggested (14, 16). Similar competition patterns between more recent kin lineages have been reported, where angiosperms ultimately outcompeted conifers from tropical dominance over the last ~350 My (63).

One supergroup that differs from the general pattern of slow but steady initial diversification is Archaeplastida. Our analyses indicate that Archaeplastida experienced a fast and early diversification throughout the Proterozoic, dominating the phylogenetic diversity of crown eukaryotic communities during this period. We postulate that this early success of Archaeplastida is the result of the cyanobacterial endosymbiosis in an ancestor of this group, responsible for establishing the first photosynthetic organelles (plastids) and thus for giving eukaryotes the transformative capacity to use light to produce chemical energy (64, 65). The rapid diversification of Archaeplastida may even be seen in the fossil record, with putative archaeplastid fossils identified during the late Paleoproterozoic (14, 15), which are potential representatives of stem Archaeplastida (15) and then the appearance of crown groups by the latest Mesoproterozoic. In this context, we further hypothesize that the early success of Archaeplastida reached far beyond the limit of this group, ultimately impacting the evolution of all photosynthetic eukaryotes. From archaeplastid algae, photosynthetic organelles were transferred by several rounds of eukaryote-to-eukaryote endosymbioses (22) to other crown groups that we infer were slowly diversifying during the Proterozoic. Supergroups such as Cryptista, Haptista, and to a lesser extent Alveolata and Stramenopila show relatively small pulses of diversification rates throughout the Proterozoic, perhaps a phylogenetic record of the acquisition of plastids in these groups.

Archaeplastida might have also impacted early on nonphotosynthetic eukaryotic groups. Our analyses reveal high phylogenetic diversity in Rhizaria and Discoba (or Amoebozoa, depending on the rooting scenario) throughout the Proterozoic, which are supergroups that use a wide variety of heterotrophic strategies from active predation to parasitism. Recent fossil evidence suggests increasingly complex ecological dynamics, including ectosymbiosis by 1000 Ma (66) and eukaryovory as far back as ~1750 Ma (9). Ovoid perforations found in organic-walled assemblages from 1150 to 900 Ma are interpreted as traces of selective predation (67), biomineralized scales ca. 810 Ma are interpreted as defense against predation (68), and direct evidence of predators themselves can be found in the first testate amoebae at ca. 740 Ma (69, 70). It is therefore possible that the early divergence of the heterotrophic lineages Rhizaria, Discoba, and Amoebozoa was spurred in part by the earlier expansion of Archaeplastida, perhaps used as prey along other members of the community. The slow but episodic oxygenation of the deep ocean (71), may have also played a role in the expansion of these heterotrophic lineages since a large proportion of environmental DNA associated to extant Rhizaria, Discoba (more precisely Euglenozoa), and Amoebozoa are found exclusively in the deep ocean and sediments (72).

## Conclusions

The early evolution of eukaryotes remains poorly understood, owing in part to the sparsity of the Proterozoic rock and fossil record (i.e., ref. 73), and difficulties in associating many early Proterozoic fossils with present day taxonomic groups. At the same time, molecular data have remained too scarce for the uncultured majority of microbial life to better represent the vast diversity of eukaryotes, at least for applying phylogenetic and diversification models. Here, we take a significant step toward resolving these challenges by integrating environmental diversity data with richer phylogenetic signals into a diversification framework spanning all major eukaryotic supergroups. Even so, our estimates based on this large dataset suggest that we may have captured as little as half of the total eukaryotic diversity, underscoring how much more hidden diversity awaits discovery. This is of course a limitation imposed by current environmental sampling, but this limitation is in part alleviated by the use of diversification methods that account for partial sampling. Our broadscale analyses indicate that crown group eukaryotes were not just present in the Proterozoic, but diversifying and probably actively engaging in ecological interactions that may have promoted further their diversification. Therefore, the geologically stable and fossil-poor billion-years-period spanning the late Paleoproterozoic to early Neoproterozoic, sometimes referred to the “boring billion,” was in fact biologically exciting (16, 74–76) witnessing a period of steady diversification that ultimately resulted in the ecological rise of crown eukaryotes.

## Material and Methods

All scripts, raw files, resulting phylogenetic trees, and other resources needed for the replication of the analyses performed and presented in this study are publicly available at ZENODO (10.5281/zenodo.17901848) and explained in detail in github.com/MiguelMSandin/EarlyDiverEuk.

### Dataset Assembly.

The Protists Reference Ribosomal database [PR2; (77); version 4.14] of the 18S ribosomal DNA (rDNA) was combined with a recently published dataset of the near-full length rDNA [18S + 28S rDNA; (78)] obtained from long-read sequencing environmental samples through Circular Consensus Sequencing by Pacific Bioscience (referred to as PacBio dataset from here on). In order to avoid redundancy, the 183,237 nuclear eukaryotic sequences of the PR2 database were clustered at 99% Operational Taxonomic Units (OTUs) with Mothur v1.44.1 (79), resulting in 59,510 OTUs. The two datasets were compared for identical sequences with VSEARCH v2.14.1 (80) and we removed the shortest identical sequence to maximize phylogenetic information. In total, 59,154 sequences of the PR2 database and 16,821 from the PacBio dataset were assembled together into 75,975 nonredundant OTUs of the rDNA.

We have additionally considered the global dataset of the V4 hypervariable region of the 18S rDNA [EukBank: (81)] to increase richness diversity into our analyses. In total, 335,662 metabarcodes of the V4 hypervariable region of the 18S rDNA gene were compared against our previously assembled dataset for similar sequences with VSEARCH v2.14.1 (80). Of all metabarcodes, 10,717 (3.2%) were fully overlapping with the previously assembled dataset, 40,286 (12.0%) were at least 99% similar, 99,565 (29.7%) were at least 97% similar and 95,350 (28.4%) were at most 80% similar. Given i) the relatively high number of metabarcodes with low pairwise similarity, ii) their low phylogenetic information (357.2 ± 60.2 average base pair length), iii) the difficulties to analyze phylogenetic placement of distant metabarcodes at global eukaryotic scale (82), iv) the ambiguities on the nature of high-throughput short metabarcodes [i.e., sequencing errors vs. intragenomic variability: (83, 84)], and v) a trade-off between sufficient diversity coverage and keeping computational efforts technically feasible, we decided not to include this dataset in our analyses. This apparent low overlap between datasets (i.e., 3.2% of exact matches) is intriguing and may result from several well-known methodological issues such as primer bias, sequencing depth and errors, or biological effects such as localized specific biogeography (e.g., populations), intragenomic variability, and/or genuine diversity stochastically escaping sampling.

### Build Initial Constraint Tree.

Initial constraint tree (*SI Appendix*, Fig. S1) was obtained from the EukProt database (85) and complemented with the latest phylogenomic studies within specific supergroups: Amoebozoa (86), Alveolata (87), Archaeplastida and Cryptista (88–90), Centrohelida and Haptista (91), Nucletmycea (92), Opisthokonta (93), Rhizaria (94), Stramenopila (95), Telonemia (96), and other orphans groups (97) such as Hemimastigophora (98) and Malawimonads (99). When specific nodes showed contrasting topologies in different studies or low support, the given node was not resolved in the initial constraint tree and was left as a polytomy. Specific nodes relevant for fossil calibration were further resolved based on comprehensive phylogenomic and/or phylogenetic studies. These nodes are Bilateria (100, 101) within Opisthokonta; Ciliophora (102) and Dinoflagellata (103) within Alveolata; Radiolaria (104) and Foraminifera (105) within Rhizaria; and Ochrophyta (106) within Stramenopila. The constraint tree was optimized over several phylogenetic analyses to avoid constraints generating polytomies or near-0 branches. The final constraint tree used for downstream analyses was obtained by replacing the given clade name (from *SI Appendix*, Fig. S1) by all the sequences bearing such name in a common polytomic node (described in detail in the github repository and available in ZENODO).

### Progressive Phylogenetic Reconstruction of >75,000 Taxa.

Phylogenetic analyses were performed in three steps over the 75,975 nonredundant eukaryotic OTUs of the rDNA with the constraint tree built in the previous section:

-The first step was aimed to build a solid backbone phylogenetic tree containing OTUs biologically relevant. Such OTUs were defined as representatives of at least 10 other sequences (for the PR2 database) or with at least 10 reads (for the PacBio database). Specific eukaryotic groups with a low representation in databases or environmentally scarce yet holding a valuable phylogenetic position were manually added to the previous selection of OTUs. These groups were Ancoracystida, Cephalochordata, Filasterea, Glaucocystophyceae, Gromia, Hemichordata, Katablepharidaceae, Malawimonadidae, Mantamonadida, Mesostigmatophyceae, Monothalamids, Noctilucophyceae, Palmophyllophyceae, Parabasalia, Placozoa, Pluriformea, Preaxostyla, Protalveolata, Rhodelphea, Synchromophyceae, and Tubothalamea. The combined initial dataset contained a total of 11,917 OTUs (11,856 OTUs representative of at least 10 other sequences and 61 OTUs manually selected) and was automatically aligned using MAFFT v7.407 (107). The initial dataset was additionally reversed (with an in-house script “fastaRevCom.py” available at “github.com/MiguelMSandin/random/”) and both forward and reverse files were aligned to account for possible misalignment issues. Aligned datasets were trimmed with trimAl v1.4.1 (108) using a 5% gap threshold (“-gt 0.05”), resulting in 7,123 and 7,304 positions respectively for the forward and reverse alignments. In order to allow phylogenetic uncertainty, we applied four different phylogenetic analyses for both the forward and the reverse alignments using the tree generated in the previous step as a constraint topological tree of major nodes. Two thorough maximum likelihood searches were done in RAxML (109) under the GTR model of evolution (110) and Gamma and CAT substitution rates optimization models over 100 bootstraps. The option “-D” was used to stop maximum likelihood convergence criterion if the relative Robinson–Foulds distance between the trees obtained from two consecutive lazy SPR cycles is smaller or equal to 1%. Ten additional quick searches were obtained in RAxML-NG (111) with a GTR model of evolution and Gamma substitution rates optimization models. The two best scoring trees were kept for downstream analyses in order to allow a greater phylogenetic uncertainty of deep and ancient nodes. In total, eight backbone phylogenetic trees were obtained at this step: RaxML GTR + Gamma, RaxML GTR + CAT, and two runs in RaxML-NG for both the forward and reverse alignment. Resulting backbone phylogenetic trees of this step were processed to remove long branches using the function “prune” from the package Bio.Phylo (112); implemented in the in-house script “treePruneOutliers.py”). Briefly, long branches were determined by identifying outliers from a normal distribution (applying the generalized Extreme Studentized Deviate, gESD, method from ref. 113).

-The second and third steps were aimed at completing the phylogenetic trees based on biological relevance, so OTUs representatives of at least two other sequences or with at least two reads (in total 36,629 OTUs) were used in the second step and all remaining OTUs (in total 75,975 OTUs) in the third step. Datasets containing the sequences were aligned and trimmed as described earlier (for both the forward and reverse datasets). Final aligned datasets had 6,705 and 6,691 positions in step 2 and 5,845 and 5,794 positions in step 3 respectively for the forward and reverse alignment. Pruned trees from the first step were used as a constraint topological tree (without branch-lengths) to infer phylogenetic trees in the second step in IQ-Tree v2.0.3 (114) under the GTR+Gamma+Invariant sites evolutionary model and including the “-fast” option. Each phylogenetic analysis was run with two replicates to allow phylogenetic uncertainty, resulting in 16 total phylogenetic trees at step 2. Resulting trees from step 2 were pruned from long-branches (following step 1) and from intruders with the in-house script treeCheckIntruders.py’. Briefly, intruders were defined as those taxa identified under a specific supergroup and phylogenetically resolved within a monophyletic clade of a different supergroup. Final cleaned (or pruned) trees from step 2 were used as topological constraints to infer phylogenetic trees in step 3, and using the same approach for the phylogenetic inference and processing of the trees. Step 3 resulted in a total of 32 phylogenetic trees.

Total number of initial, pruned, and final tips, as well as evolutionary model, constrained trees, and likelihoods of every tree at every step generated and analyzed in this study can be found in Dataset S2. Each final tree was rooted in Discoba and Amorphea (gathering Amoebozoa, Apusomonadida, Breviatea, Holozoa, and Nucletmycea) by creating an outgroup in the given node using the function “set_outgroup” from the package ete3 (115); implemented in the in-house script ‘treeRootOutgroup.py’.

### Phylogenetic Dating.

Resulting phylogenetic trees were time-calibrated in TreePL (116) using a total of 77 well-supported fossil calibrations across the tree (*SI Appendix*, Fig. S3 and Dataset S1). Such calibrations were chosen based on i) monophyletic nodes, ii) clades consistent among the 32 phylogenetic trees, iii) at least four OTU representative sequences in all phylogenetic trees, iv) consistency between branch-lengths and calibration age, and v) the calibration has been previously validated in clade-specific molecular clock analyses. In order to allow uncertainty in calibration of the phylogenetic distance, TreePL was ran over 100 replicates on each tree, including the options “thorough” and “log_pen.” Final dated trees were obtained with treeAnnotator v1.10.4 (117) by summarizing the node height to the median value. Branch lengths of the time-calibrated trees were transformed to millions of years (Ma) with the R package “ape” (118) for following analyses. Here, we have applied stringent and conservative criteria for fossil selection and calibration in order to minimize potential sources of uncertainty for each individual fossil calibration [i.e., interpretation of the fossil record and further attribution to specific nodes in the phylogenetic tree; (119, 120)], yet many more fossil calibrations could potentially narrow down the posterior density.

We additionally calibrated 10 phylogenetic trees randomly chosen in an attempt to accommodate recent findings and reinterpretations of the fossil record. The first additional calibration set (referred to as Molecular Clock 02: MC02) was chosen to include the potential early Rhodophyta fossil *Rafatazmia* at 1600 Ma old (39), used as a minimum constraint. Yet its taxonomic assignment (40) and age (18) have been questioned and therefore not considered in our primary calibration set (referred to as MC01). The second additional calibration set (referred to as MC03) considers *Bangiomorpha* as a potential stem Rhodophyta (40), instead of a member of Bangiophycea (18) as in MC01. Here, we applied *Bangiomorpha*’s calibration to its closest node in crown Rhodophyta, as in ref. 21, since molecular phylogenetics cannot resolve stem groups. A third additional calibration set (MC04) was applied to take into account uncertainties surrounding the first diatom fossils (41), delaying the first appearance of this clade from ~190 Ma (as a minimum constraint) to ~125 Ma (as a minimum appearance time in the fossil record). Therefore, in order to allow ambiguity in the calibration of diatoms, we produced a third calibration set (MC04) without the crown diatom calibration.

### Lineages Through Time.

As an approach to summarizing the time-calibrated trees, we computed Lineages Through Time (LTT) plots directly from the time-calibrated trees obtained in the previous section (“Phylogenetic dating”) with the in-house script “treeLTTsubTrees.R.” LTT were used and only used for the purpose of summarizing complex time-trees. Indeed, LTT lack all lineages that did not leave any descendant in the present and can therefore only be interpreted in terms of diversification by using birth–death diversification models. For example, in the presence of extinctions, LTT can give a spurious impression that diversification accelerates [the “pull of the present” effect; (26)], caused by the fact that extant, young lineages have not had time to go extinct. Supergroups were extracted with the function “tree_subset” from the R package “treeio” (121). These analyses, as well as the ones that follow (“Diversification analyses”) were carried out directly and independently on each time-calibrated tree of eukaryotic supergroups with at least 300 tips.

### Diversification Analyses.

#### Estimation of the diversity.

Fitting diversification models to time-calibrated phylogenetic trees requires estimating the fraction of the total diversity that the analyzed tree represents. Such an estimate (hereon referred to as “sampling fraction”) represents the probability that a given taxa is included in the tree. However, the estimation of the total number of eukaryotic species varies dramatically among studies, ranging from 1.8 to 2 million species (122) to 5 ± 3 million species (123), 8.7 ± 1.3 million species (124) or even up to 10^11^ to 10^12^ total microbial species (125). Far from settling this debate, here we refer to species as 99% OTUs of the 18S rDNA gene sequences avoiding any further ambiguity in the species criterion (see e.g., refs. 126 and 127 for further details on the so-called species problem). While different species criteria cannot be directly compared, recent efforts to unify global metabarcoding data resulted in 335 662 OTUs of the V4 hypervariable region of the 18S gene (81) or ~40,300 OTUs of the V9 hypervariable region of the 18S gene (128), agreeing with our study in a similar order of magnitude of observed OTUs (75975 OTUs of the 18S rDNA). A global survey of eukaryotic plankton diversity of the sunlit ocean (129), estimated a total eukaryotic plankton richness of ~150,000 OTUs from ~110,000 sampled OTUs (covering ~73% of the total diversity). In this context, we estimated different sampling fractions to provide a minimum and a maximum scenario allowing and acknowledging ambiguity in the total number of species.

Sampling fractions were estimated on all supergroups of eukaryotes composed of at least 300 tips. We applied both the Preston’s Lognormal Model (130) and the truncated lognormal model, implemented in the R package “vegan” (131) to search for maximum and minimum boundaries of the sampling fraction. To account for differences in abundance between the two datasets (i.e., PR2 and PacBio), reads were normalized at 1,000, which corresponds to the closest power of 10 of the smaller maximum number of reads among the two datasets. We further assess the goodness-of-fit of the resulting models to the observed data using two independent approaches: the Kolmogorov–Smirnov (KS) statistic and the Quantile–Quantile plot (QQplot) slope. Briefly, the KS statistic measures the distance between observed and fitted values (e.g., the closer to 0, the better fit) and was computed with the “gofTest” function from the R package “EnvStats.” The QQplot compares the quantiles of two distributions (being x = y when the two distributions are identical, or a slope = 1) and was computed in base R with the functions “lm” and “sort” (i.e., ‘*lm(sort(fitted) ~ sort(observed))$coeff[[2]]*’).

#### Rates of diversification through time.

The first approach to gain insights in the early diversification of eukaryotes applies ClaDS to estimate the branch-specific rates of speciation, using the most recent implementation with Data Augmentation (44). ClaDS was chosen because the underlying model assumptions account for rate heterogeneities across the tree, as is expected within our trees covering a wide diversity. We used the version of ClaDS with constant turnover (turnover rate = extinction rate/speciation rate: “ε = μ/λ”; ClaDS2) (45). This model does not account for mass extinctions (i.e., periods when the extinction rate would be especially high). Preliminary analyses [using the CoMET model; (132, 133)] showed low support for the presence of mass extinctions, which can either be real, or linked to the known difficulty of detecting mass extinction events from extant-only data. Mean net diversification Rates Through Time (RTT; net diversification rate = speciation rate − extinction rate: “r = λ − μ”) and estimated number of LTT (or Diversity Through Time; DTT) were estimated from the reconstructed time-calibrated trees under the ClaDS module. ClaDS analyses were performed on all time-calibrated trees and both for the minimum and maximum sampling fractions. By running the analyses with both sampling fraction estimates, we quantify the effect of uncertainty in these estimates on our diversification results. This provides a plausible range of possibilities, since maximum likelihood diversification rate estimates are monotonous functions of the sampling fraction (i.e., the lower the sampling fraction, the more missing lineages, and the higher the estimated diversification rates and diversity) and thus, intermediate sampling fractions would provide intermediate diversification rate estimates and diversity curves.

Given the size of the time-calibrated trees, certain analyses were relatively demanding in terms of computational resources and did not finish after 30 d, four independent attempts and up to 180 GB of RAM memory. Supergroups such as Nucletmycea, Holozoa, and Alveolata were the most affected. Completed RTT and DTT from each supergroup of eukaryotes were independently summarized into single plots by estimating the median (and 90% interquartile range) of different plots per supergroup. A summary of the resulting model parameters can be found in *SI Appendix*, Fig. S11.

#### Shifts in diversification rates.

The second approach is aimed at identifying shifts in diversification dynamics by estimating speciation and extinction rates with the BAMM (134) package on the time-calibrated trees, over both the minimum and maximum sampling fractions, and following the recommendations described in the online documentation (bamm-project.org/). Briefly, speciation and extinction rates are modeled within rate regimes using an exponential change function. Priors were estimated with the function “setBAMMpriors” from the R package BAMMtools (135), and the control file was generated with the function “generateControlFile” setting four MCMC chains for 10 million generations and sampled every 100 steps (for Alveolata, Holozoa, and Nucletmycea MCMC chains were run for 100 million generations sampled every 1,000 steps). The most plausible number of speciation rate shifts was estimated over four eukaryotic time-calibrated trees chosen at random. To do so, preliminary analyses were run over their extracted supergroup trees with 10 and 50 expected shifts in diversification and the posterior shift probabilities were examined with the functions “computeBayesFactors” and “plotPrior.” For each supergroup, the most plausible number of expected shifts in configuration was chosen as the average between the highest posterior density and the highest bayes factor (*SI Appendix*, Fig. S15). Final control files were generated as described above, including the corresponding estimated number of shifts. Final results were analyzed to extract the best shift configuration with the function “getEventData” and “getBestShiftConfiguration.”

Effective Sample Size (ESS) of the number of shifts was checked with the R package coda (136). From the supergroups analyzed, Amoebozoa, Cryptista, Discoba, Haptista, and Metamonada largely exceeded a value of 200 ESS, while the rest of the clades was above 100 ESS as a minimum. Increasing the sampling chain and/or sampling frequency would imply excessive RAM memory usage in downstream analyses (which is up to 350 GB of RAM memory for ~4 d in some Holozoa trees). Selected shifts in speciation were summarized along the temporal dimension for each supergroup and estimated into a shift toward lower speciation rate or a shift toward a higher speciation rate.

## Supplementary Material

Appendix 01 (PDF)

Dataset S01 (XLSX)

Dataset S02 (XLSX)

## Data Availability

DNA alignments, phylogenetic trees, scripts and resources to replicate the results presented in this study. Data have been deposited in Zenodo (10.5281/zenodo.17901847) (137).
