# Supplementary material for: Environmental phylogenetics supports a steady diversification of crown eukaryotes starting from the mid-Proterozoic
Source: Proc Natl Acad Sci U S A. 2026 Jul 16;123(29):e2600283123. doi: 10.1073/pnas.2600283123 (PMC13389600; doi:10.1073/pnas.2600283123)
Supplement: Supplementary file 1 — Appendix 01 (PDF) [file pnas.2600283123.sapp.pdf]

PNAS

Supporting Information for

Environmental phylogenetics supports a steady diversification of crown eukaryotes starting from the mid Proterozoic

Miguel M. Sandin, Phoebe A. Cohen, Hélène Morlon, Fabien Burki

Miguel M. Sandin, Fabien Burki
Email: miguelmendezsandin@gmail.com; fabien.burki@ebc.uu.se

This PDF file includes:

Figures S1 to S15

Other supporting materials for this manuscript include the following:

Datasets S1 and S2

Figures

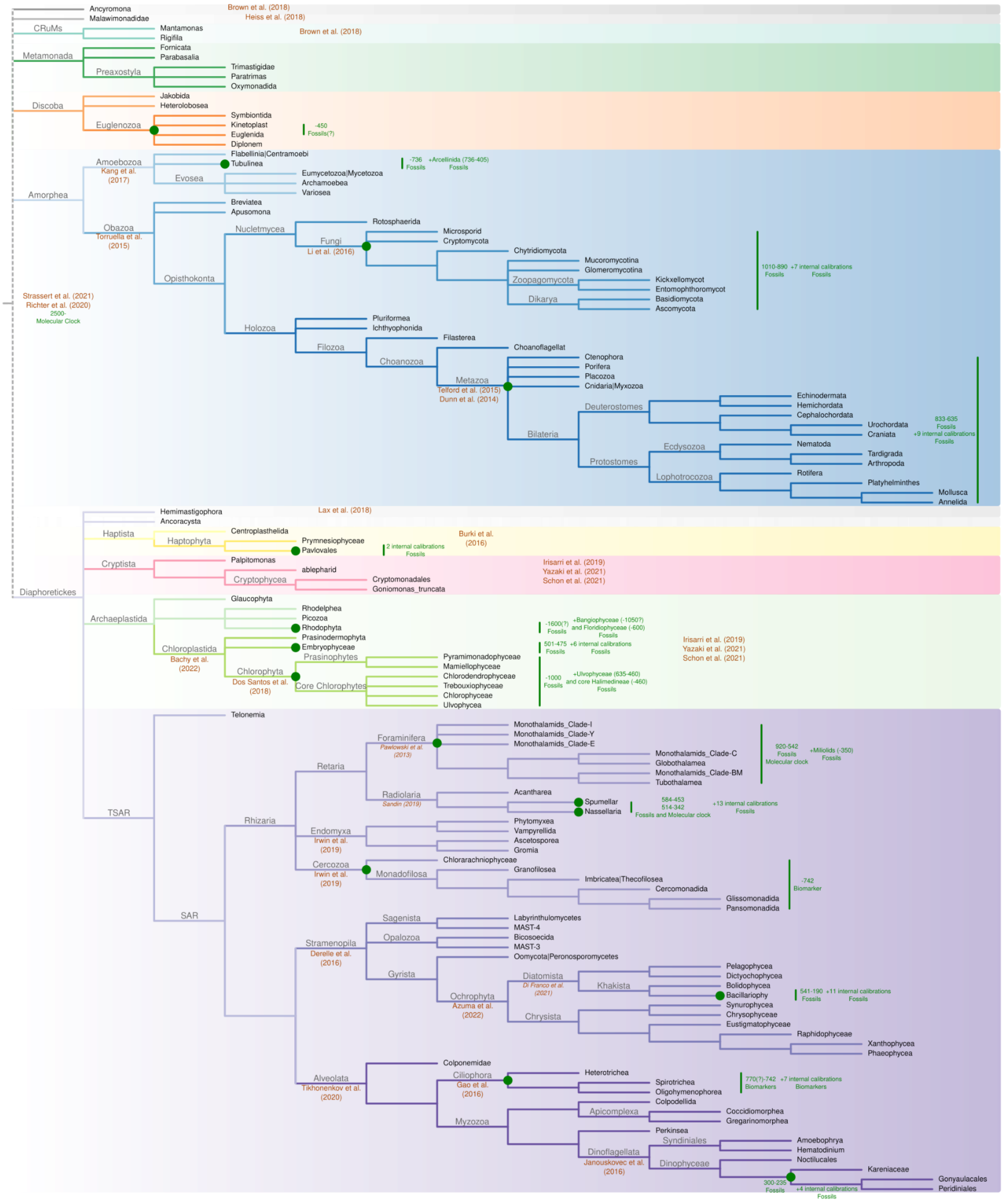

Fig. S1. A summary of the initial constraint tree used in this study. In orange are highlighted the references used at specific nodes and in green a summary of the most relevant fossil calibrations.

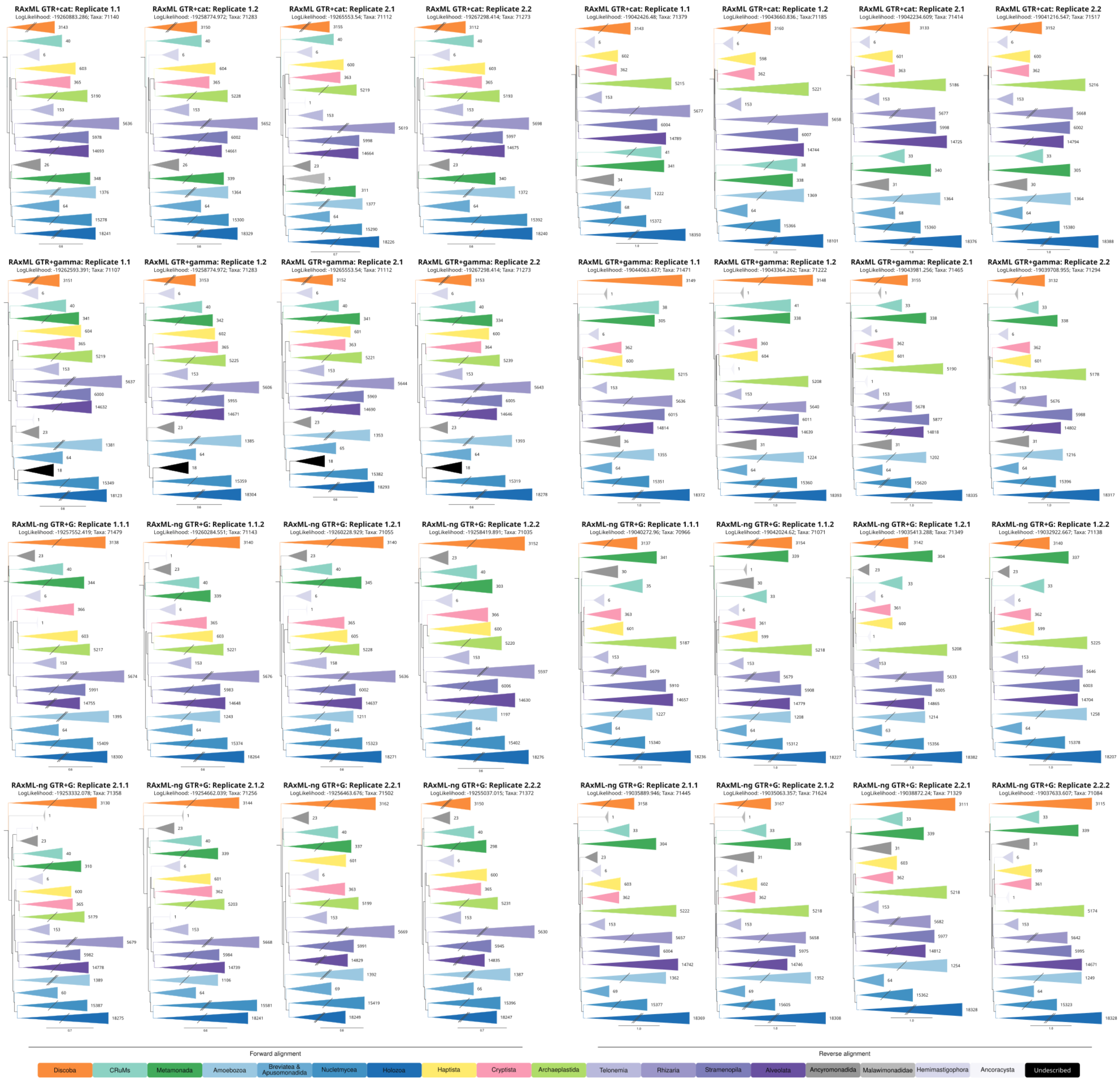

Fig. S2. A representation at the supergroup level of the different phylogenetic trees inferred and analyzed in this study rooted at Discoba.

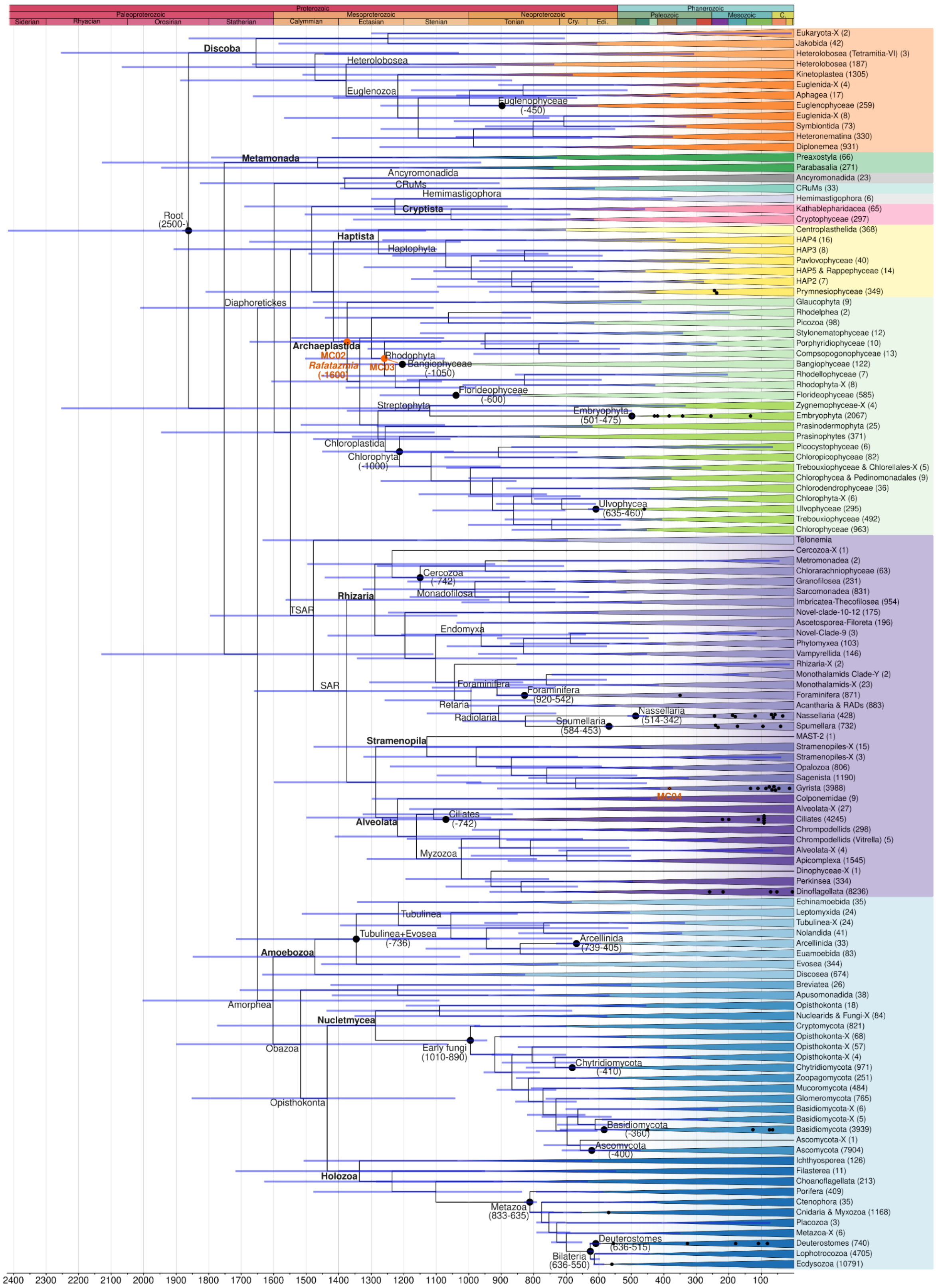

Fig. S3. All calibrated nodes over the time-tree shown in **Fig. 1A** emphasizing Proterozoic calibrations and highlighting the differences with additional calibration sets.

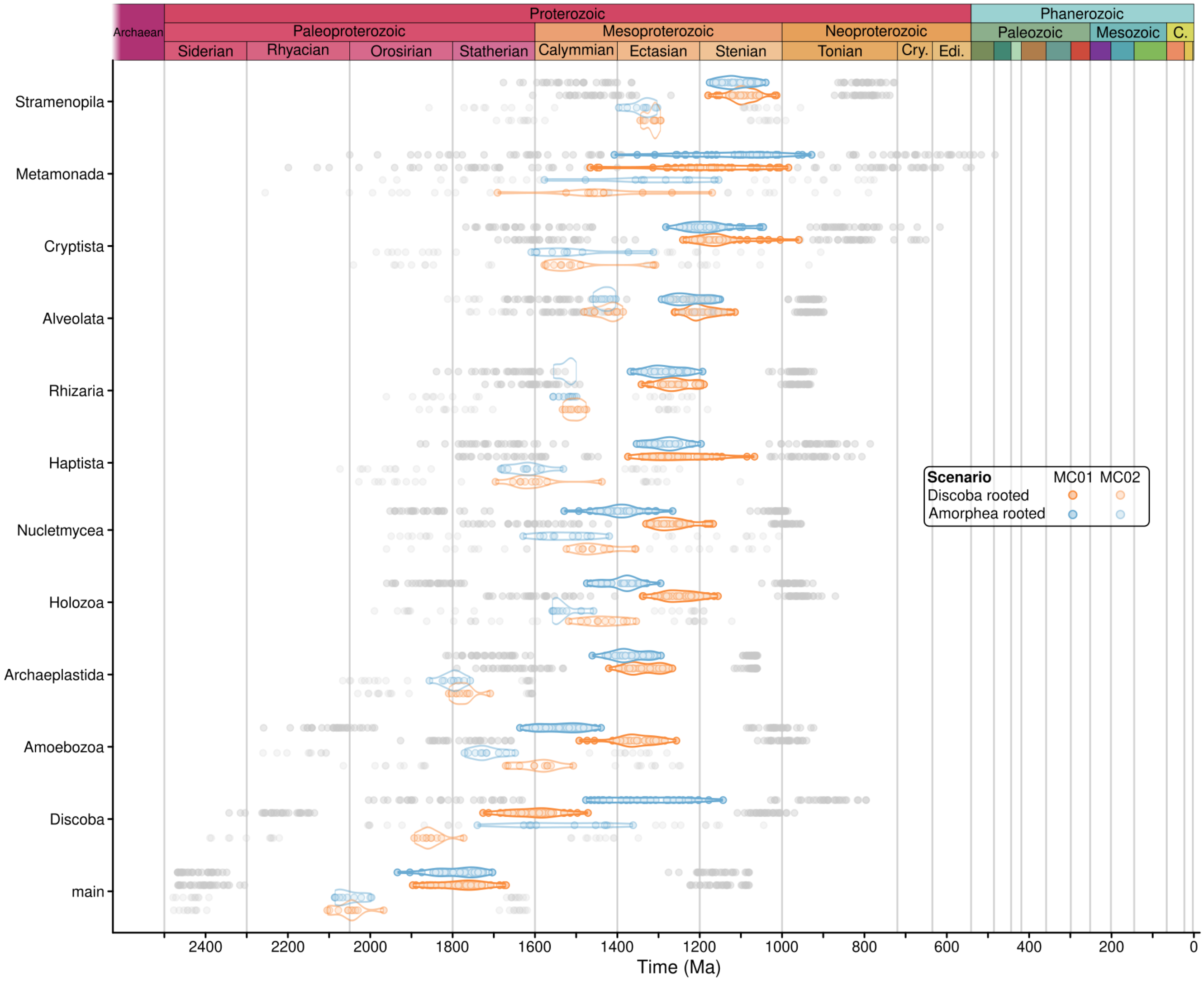

Fig. S4. Summary of median and 95% highest posterior densities of the first branching event in all time-trees generated and analyzed in this study for the two major calibration sets.

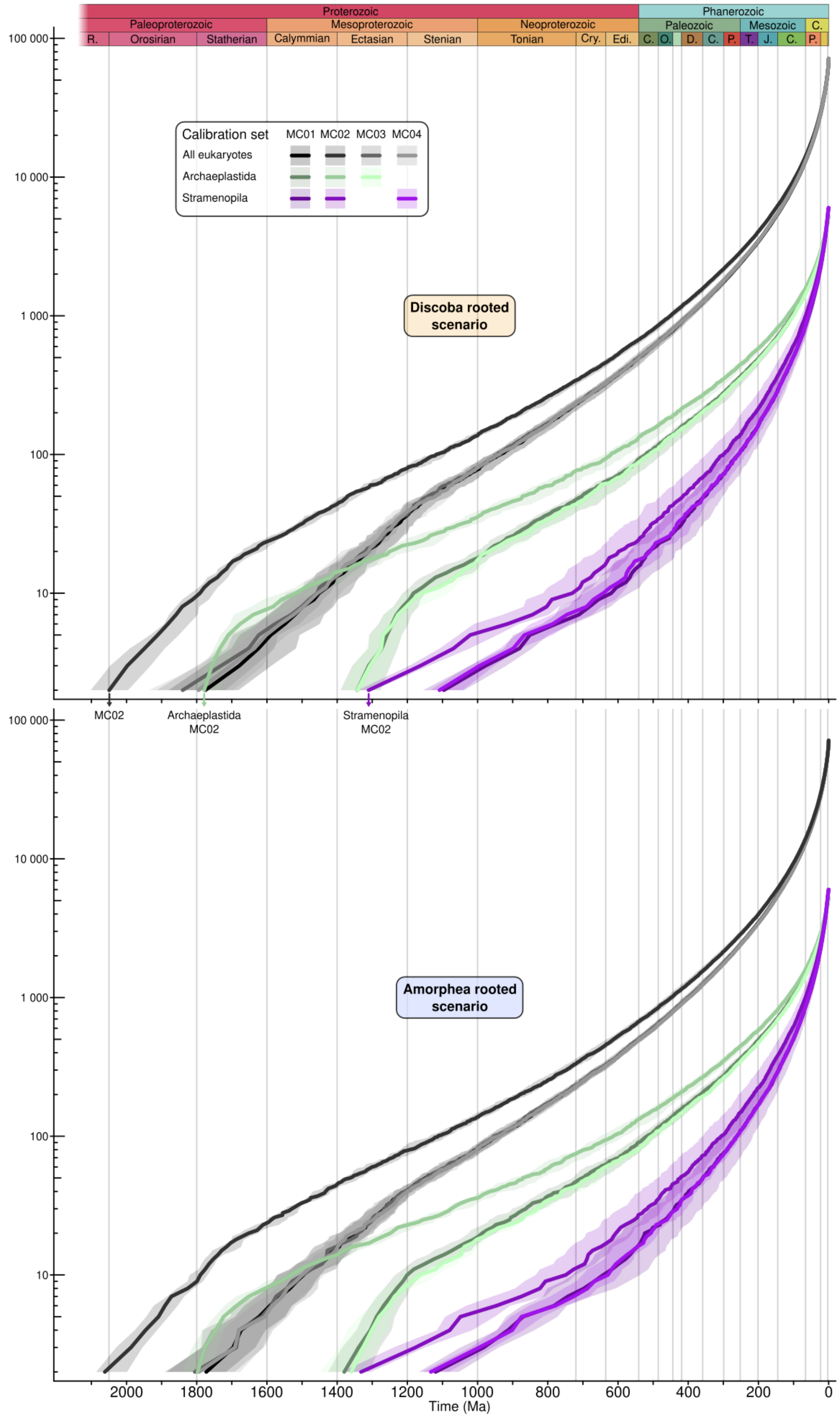

Fig. S5. Summary of the Lineages Through Time (LTT) plot of the different calibration sets on 10 randomly selected time-trees (independently for each calibration set) for all eukaryotes in different tones of gray and the supergroups containing the additional calibration points. Lines represent the median among all LTT and shaded areas represent the 90% centered percentile of the medians.

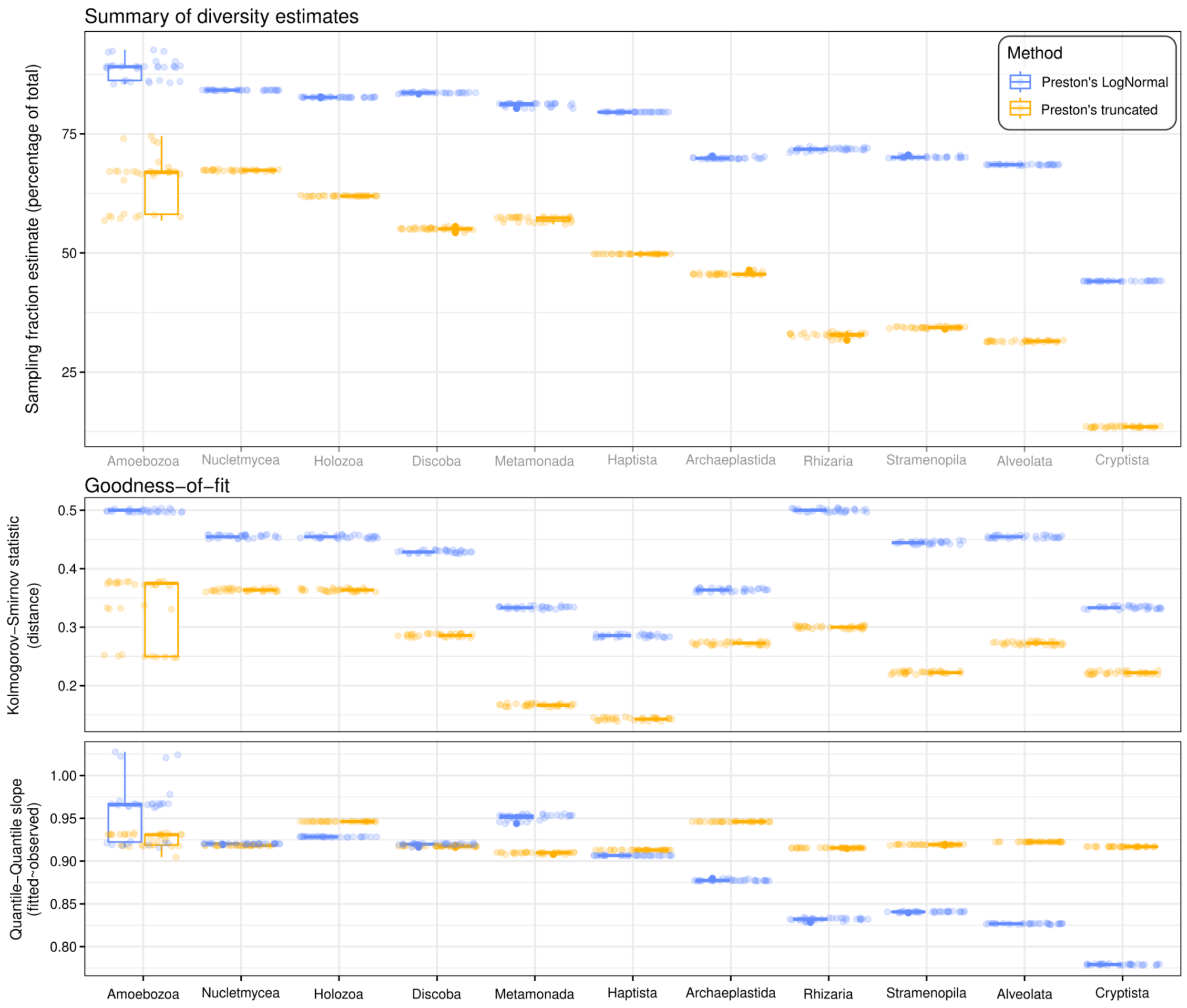

Fig. S6. Sampling fractions (given in percentage) of all eukaryotic supergroups with at least 300 tips estimated by two different approaches and the goodness-of-fit tested by two different approaches.

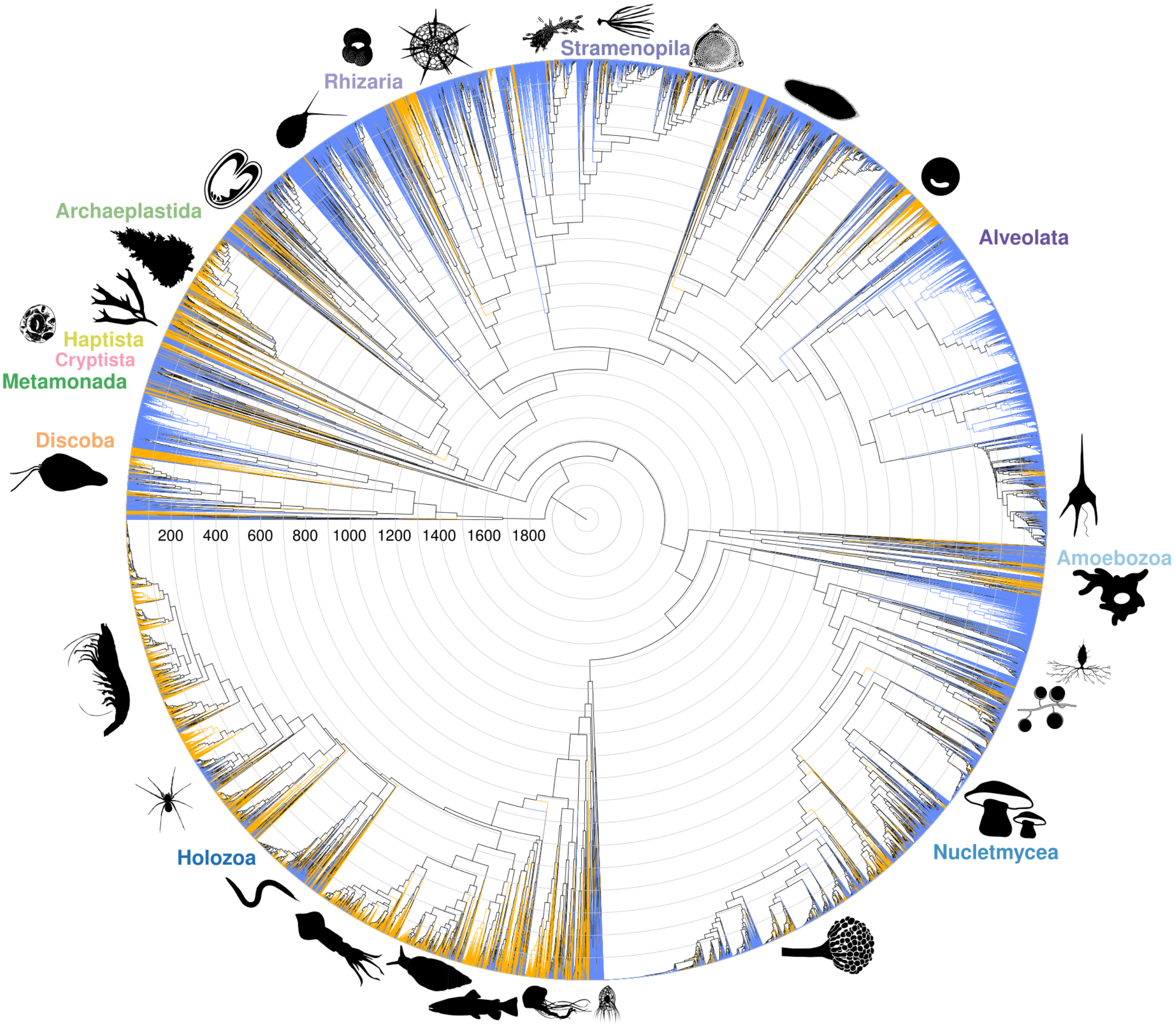

Fig. S7. The time-calibrated phylogenetic tree shown in **Fig. 1A** discriminating morphologically described OTUs (orange branches; associated to a binomial latin name, either to Genus or to Species) and environmental OTUs (blue branches; lacking morphological description). See **Fig. 1A** for further details on the figure.

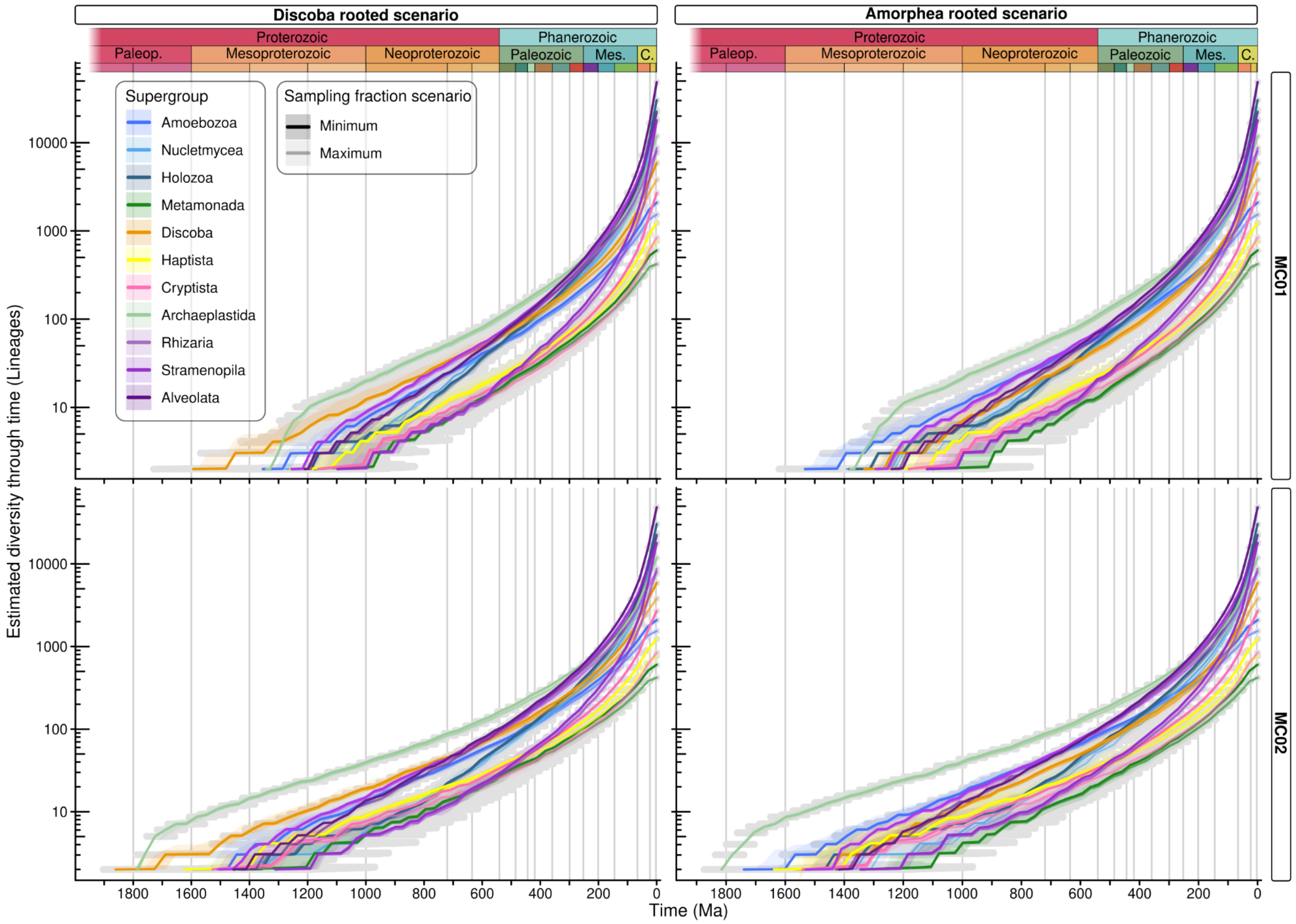

Fig. S8. Summary of the estimated Diversity Through Time (DTT) plots for the Discoba rooted scenario (left column) and the Amorphea rooted scenario (right column) when using the main calibration set (MC01, upper row) or the second calibration set (MC02, lower row). Lines represent the median, and shaded areas the median of the 90% HPD, respectively, of all independent estimated diversity through time. Gray horizontal lines represent the 90% HPD of the time scale.

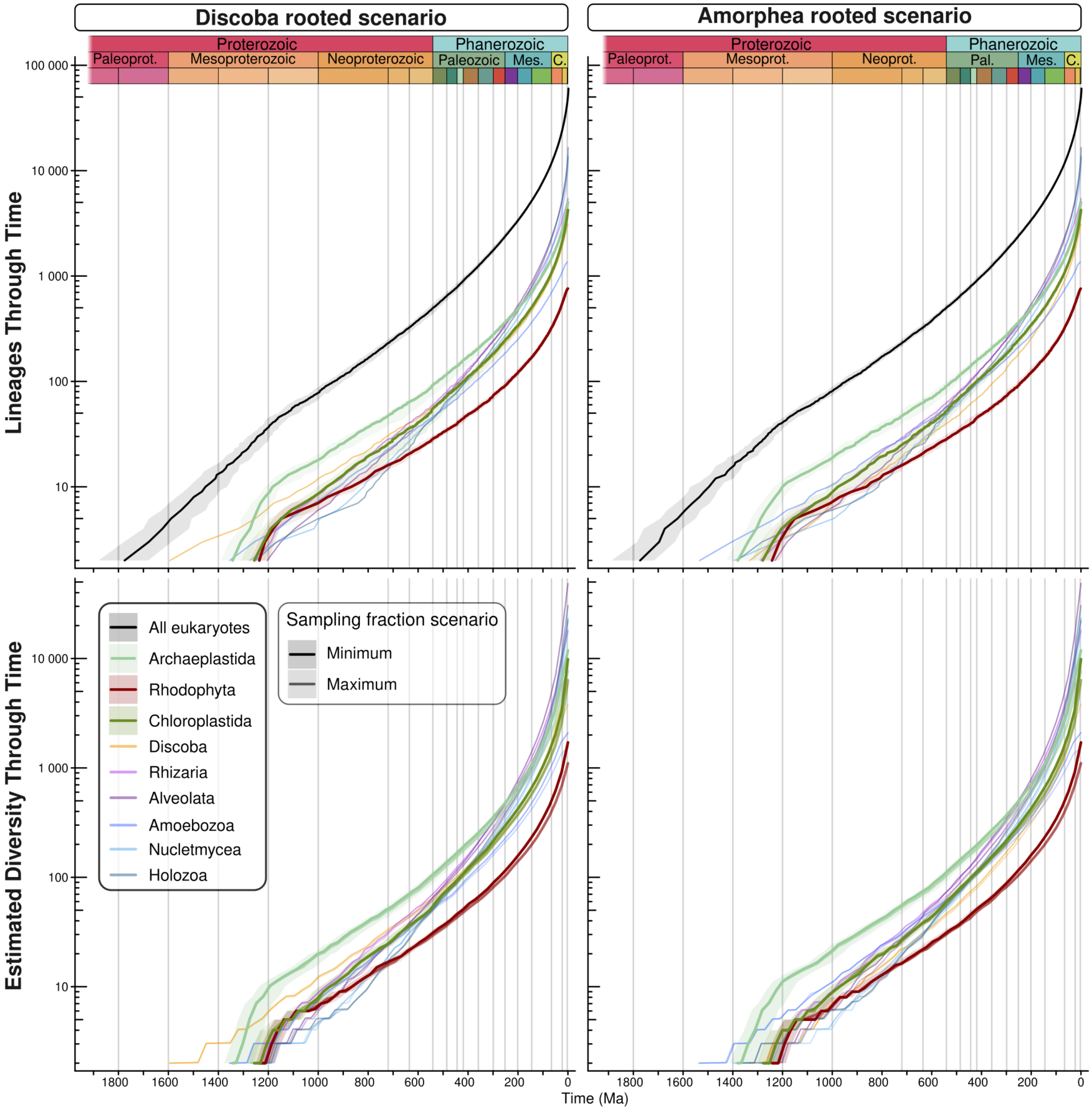

Fig. S9. Lineages Through Time (LTT) and estimated Diversity Through Time (DTT) plots shown in **Fig. 1** and **Fig. 2**, respectively, with a focus on Archaeplastida (for the Discoba -left column- and Amporphea -right column- rooted scenarios), showing the variation through time of Chloroplastida (green algae) and Rhodophyta (red algae) and that of other main supergroups of eukaryotes for comparison. Shaded area represents the 90% centered percentile of time for the LTT plots and the 90% centered percentile of the estimated diversity for the DTT plots.

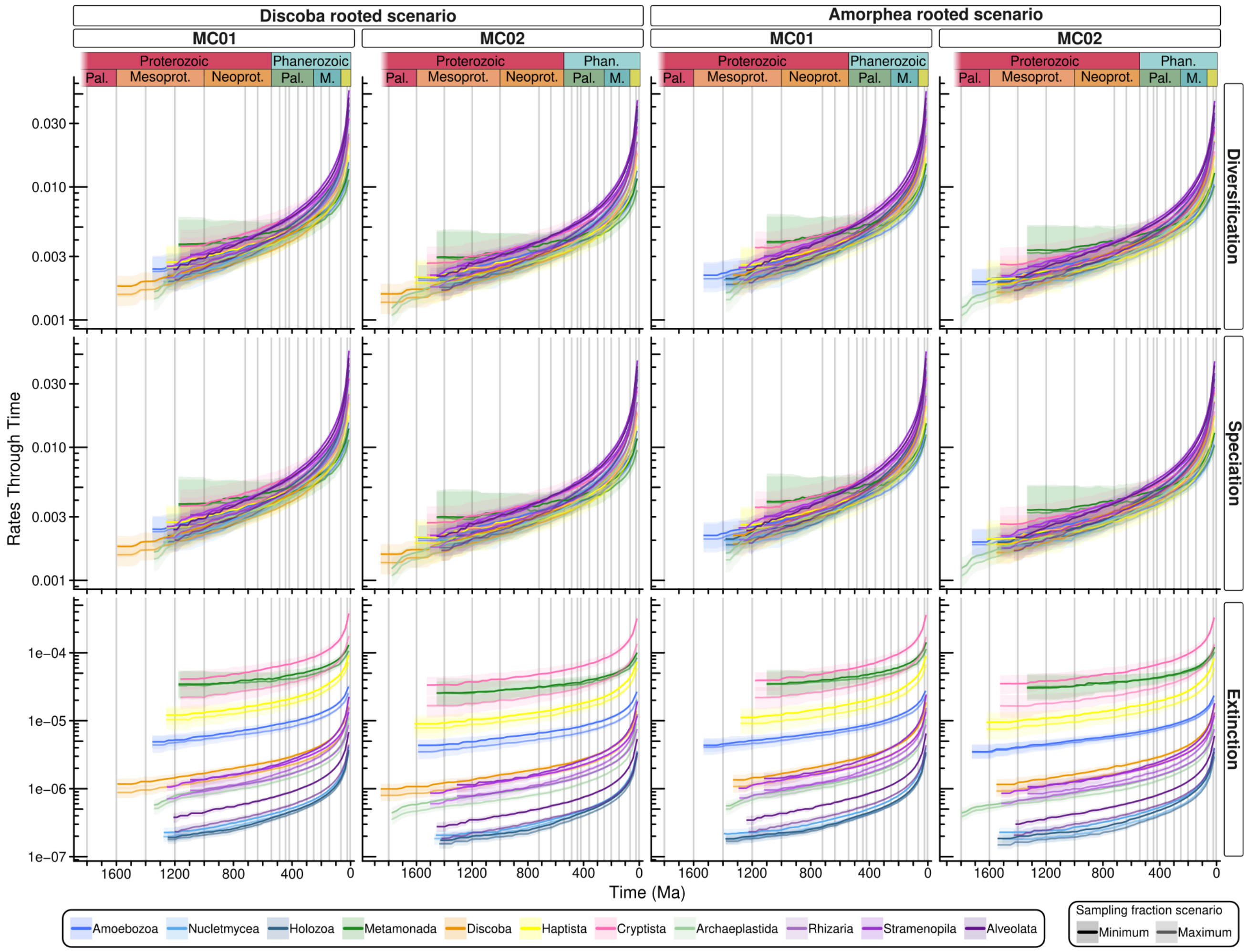

Fig. S10. Speciation, extinction and net diversification rates for the different rooting and sampling fraction (solid and transparent lines represent the minimum and maximum sampling fractions scenarios respectively) scenarios obtained from a constant turnover rate under the cladogenetic diversification rate shift model (ClaDS, with data augmentation). Lines represent the median, and shaded areas the 90% centered percentile of the median values, respectively.

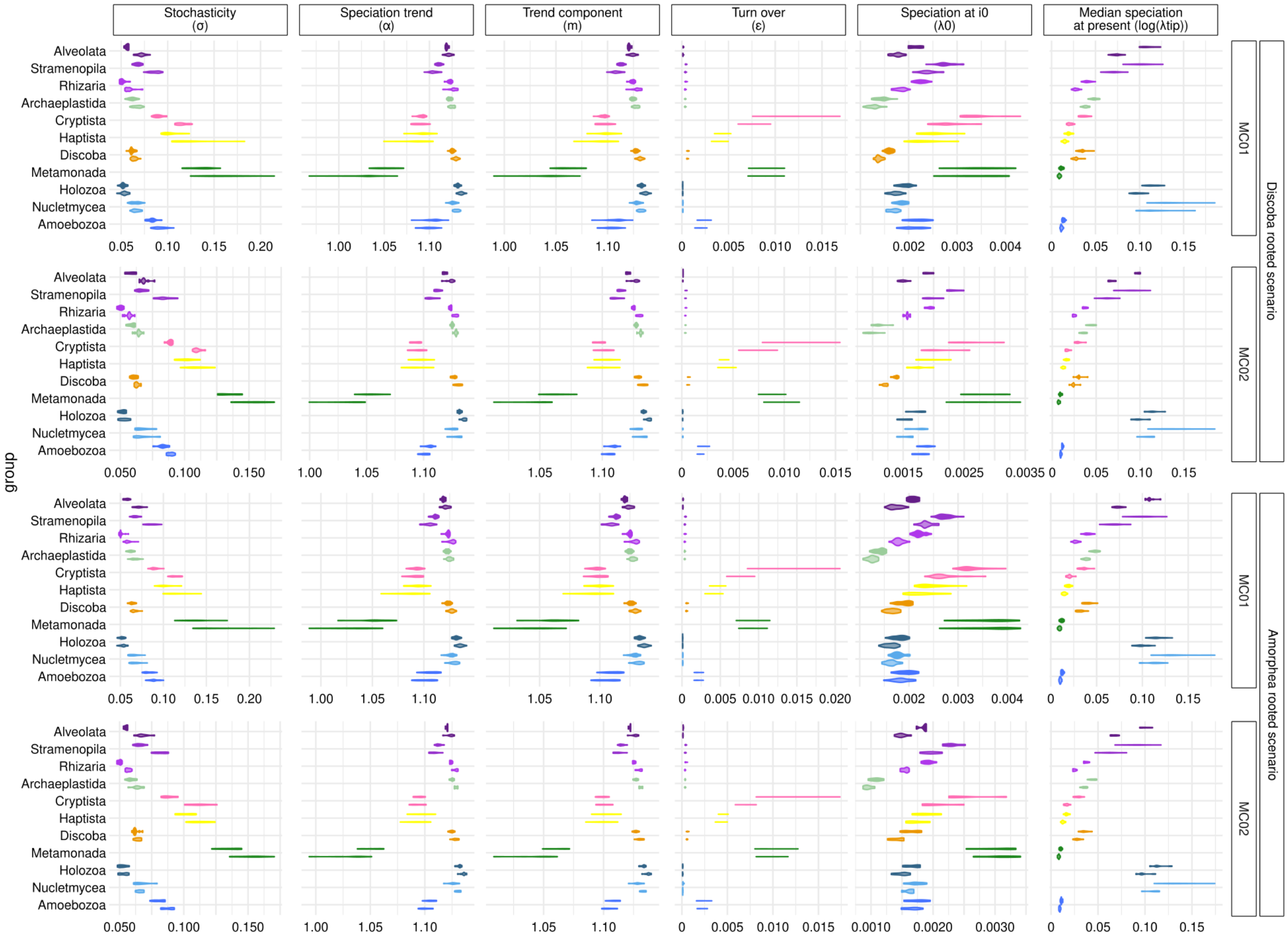

Fig. S11. A summary of ClaDS model parameters within all different scenarios and supergroups. Briefly, sigma (σ) represents the stochastic parameter of rate inheritance, alpha (α) the deterministic trend in speciation rates changes, the trend component (m=α×exp($σ^2$/2)) the average trend at speciation (whether daughter rates tend to be higher or lower than parental rates), epsilon (ε) the extinction rates at constant turn-over, Lambda0 (λ0) the speciation rate at the beginning of the process, and Lambda-tip (λtip) the median speciation rate at present-day (tips). Minimum and maximum sampling fraction scenarios are represented by solid and transparent violin plots respectively.

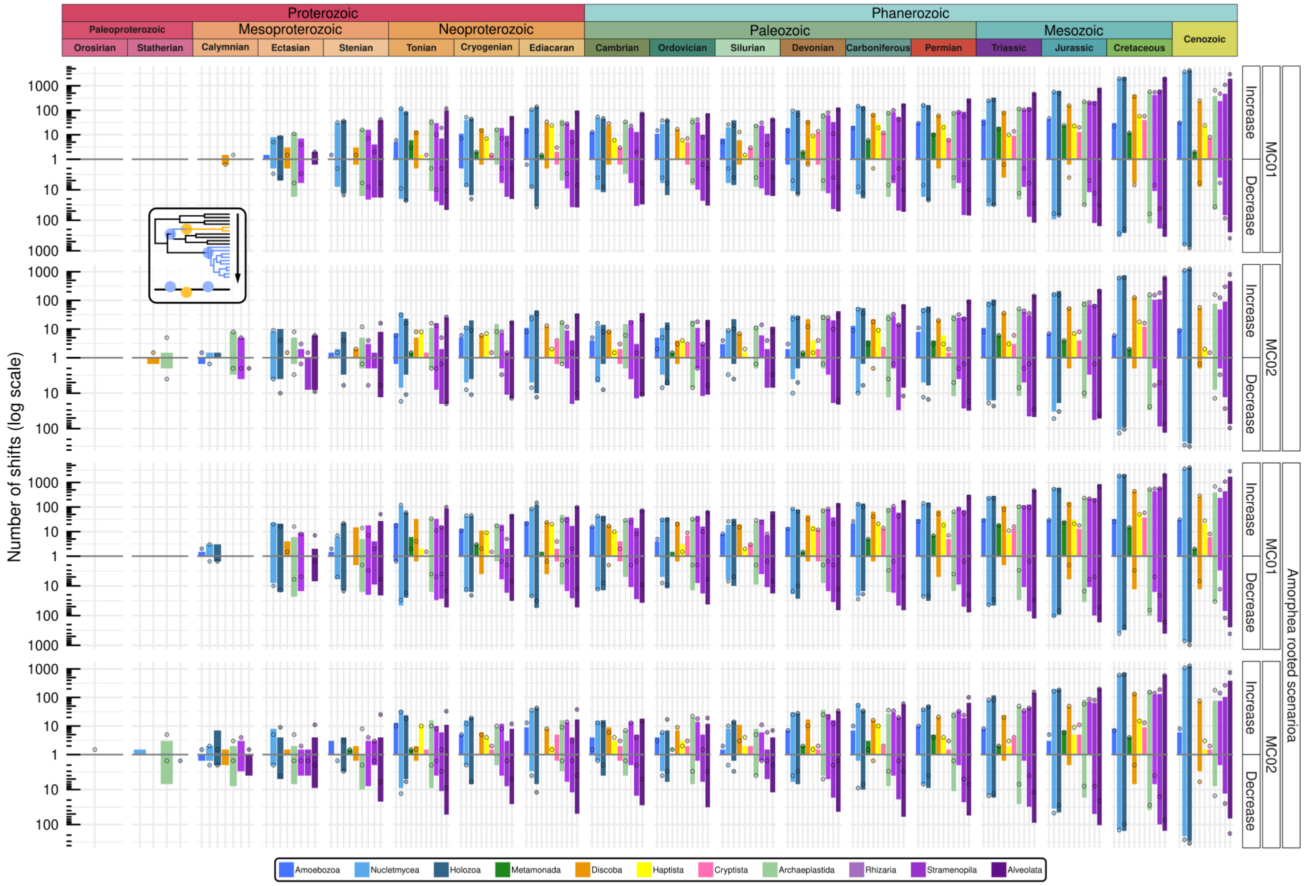

Fig. S12. Total number of speciation rate shifts up (increase) or down (decrease) for all supergroups and the two main calibration sets (MC01 and MC02) by geological period. Minimum and maximum sampling fraction scenarios are represented by solid columns and points respectively. The cartoon on the top left box exemplifies how the shift count has been obtained.

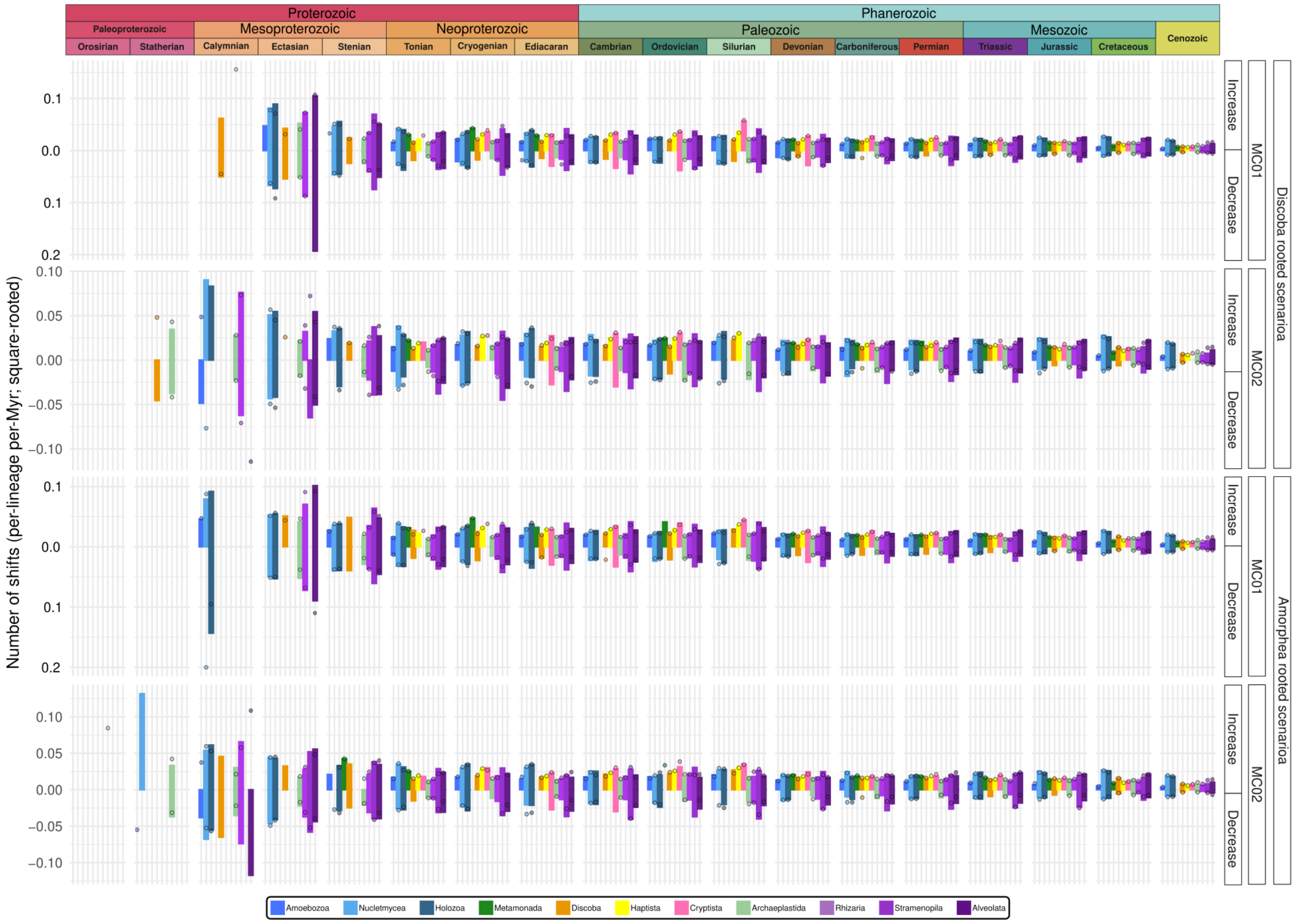

Fig. S13. Proportion of shifts in the speciation rate (square rooted) across the different geological periods, for the Discoba rooted scenario and the Amorphea rooted scenario. 'Increase' indicates a shift towards a higher speciation rate and 'decrease' indicates a shift towards a lower speciation rate. Minimum and maximum sampling fraction scenarios are represented by solid columns and points respectively. The proportion of shifts was calculated by dividing the total number of shifts per time period (**Fig. S12**) by the total branch-length within the given time period.

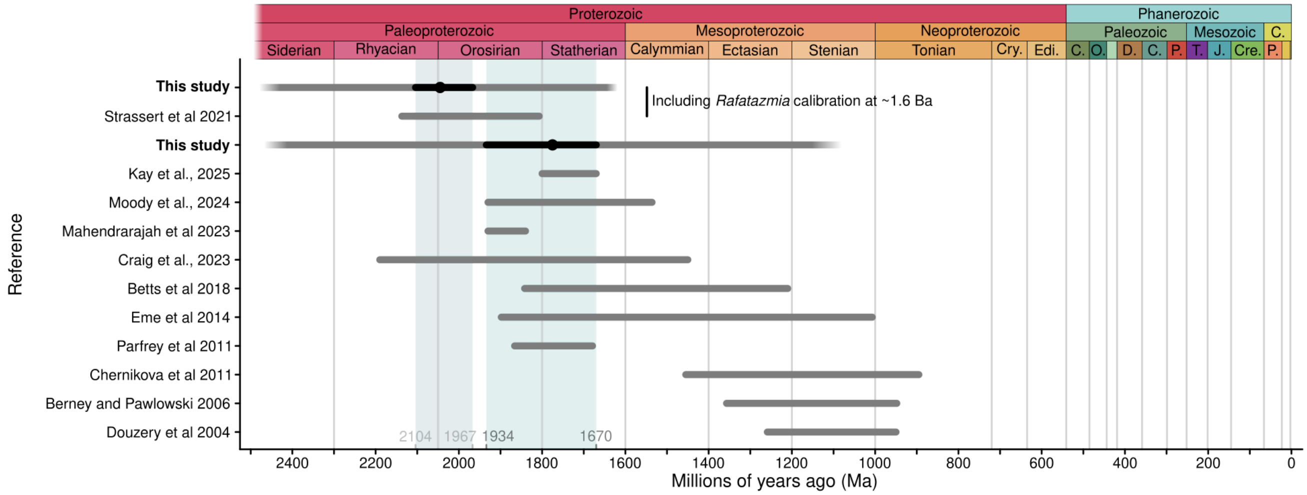

Fig. S14. A graphical representation on the estimation of the diversification of the Last Eukaryotic Common Ancestor (LECA) from different studies cited herein. Blue vertical rectangles correspond to the range estimated by our analyses, given by the black bars (the dot represents the median and the grey bars the 95% Highest Posterior Densities).

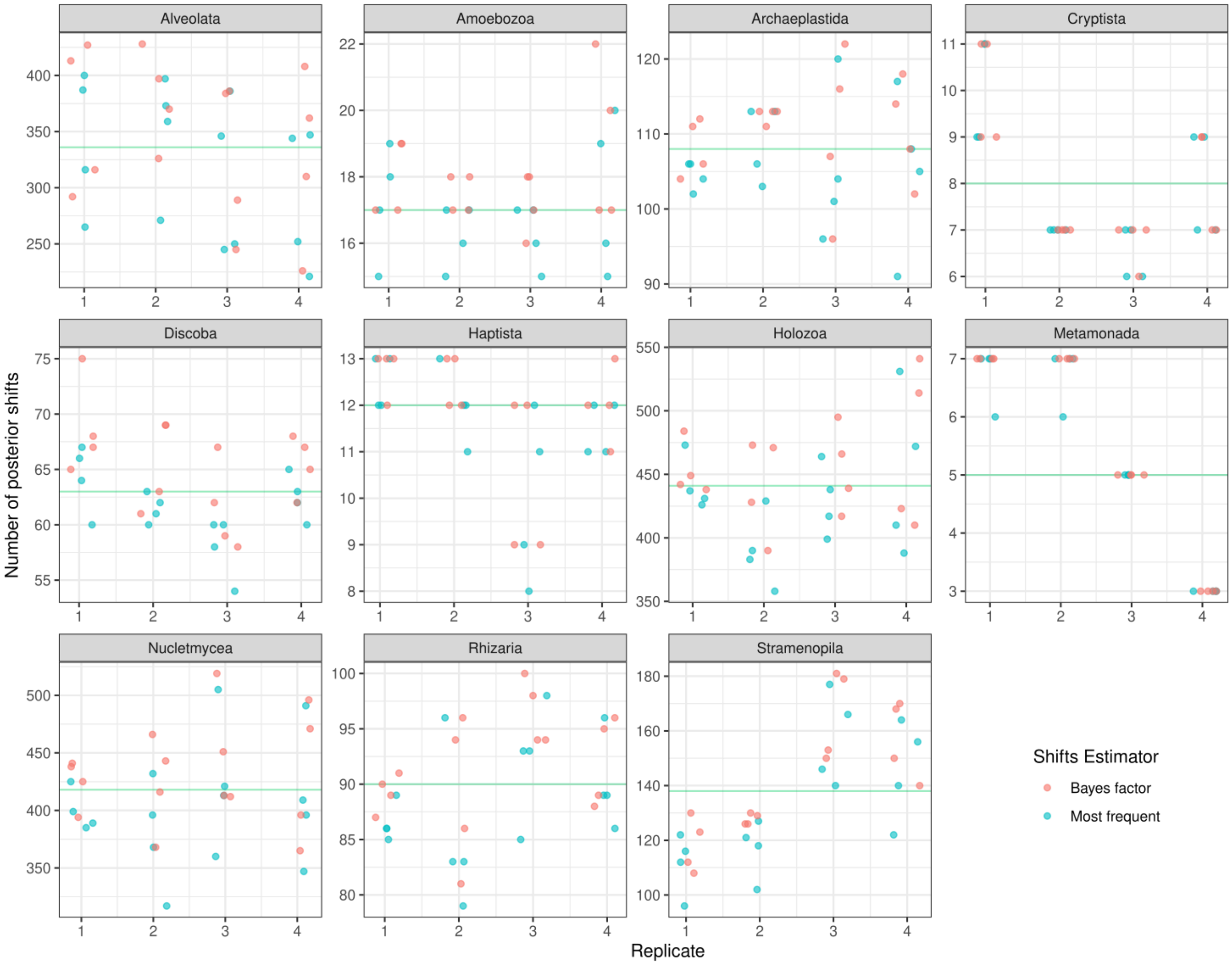

Fig. S15. Bayes factor and most frequent number of the observed number of shifts in speciation rates along the MCMC run from the BAMM analyses of all supergroups analyzed in this study.

Dataset

Dataset S1. List of all fossil calibrations used in this study.

Dataset S2. A summary of the number of branches in each step of the phylogenetic tree building and the likelihood of the trees.
